# Aortic heterogeneity across segments and under high fat/salt/glucose conditions at the single-cell level

**DOI:** 10.1093/nsr/nwaa038

**Published:** 2020-03-09

**Authors:** Dongxu He, Aiqin Mao, Chang-Bo Zheng, Hao Kan, Ka Zhang, Zhiming Zhang, Lei Feng, Xin Ma

**Affiliations:** 1 Wuxi School of Medicine and School of Food Science and Technology, Jiangnan University, Wuxi 214122, China; 2 School of Pharmaceutical Science and Yunnan Key Laboratory of Pharmacology for Natural Products, Kunming Medical University, Kunming 650500, China; 3 School of Biotechnology, Jiangnan University, Wuxi 214122, China

**Keywords:** aorta, single-cell RNA sequencing, cardiovascular disease

## Abstract

The aorta, with ascending, arch, thoracic and abdominal segments, responds to the heartbeat, senses metabolites and distributes blood to all parts of the body. However, the heterogeneity across aortic segments and how metabolic pathologies change it are not known. Here, a total of 216 612 individual cells from the ascending aorta, aortic arch, and thoracic and abdominal segments of mouse aortas under normal conditions or with high blood glucose levels, high dietary salt, or high fat intake were profiled using single-cell RNA sequencing. We generated a compendium of 10 distinct cell types, mainly endothelial (EC), smooth muscle (SMC), stromal and immune cells. The distributions of the different cells and their intercommunication were influenced by the hemodynamic microenvironment across anatomical segments, and the spatial heterogeneity of ECs and SMCs may contribute to differential vascular dilation and constriction that were measured by wire myography. Importantly, the composition of aortic cells, their gene expression profiles and their regulatory intercellular networks broadly changed in response to high fat/salt/glucose conditions. Notably, the abdominal aorta showed the most dramatic changes in cellular composition, particularly involving ECs, fibroblasts and myeloid cells with cardiovascular risk factor-related regulons and gene expression networks. Our study elucidates the nature and range of aortic cell diversity, with implications for the treatment of metabolic pathologies.

## INTRODUCTION

The aorta is the largest artery in the body, with intima, media and adventitia layers. The intima mainly contains endothelial cells (ECs) and provides a smooth surface for blood to flow through. The media, mainly muscle and elastic fibers, allows the aorta to expand and contract. The adventitia, populated by pericytes, perivascular nerves, immune cells and stromal cells, supports the structure and function of the aorta [1,2]. Recently, a study of mouse organs using single-cell RNA-sequencing (scRNA-Seq) provided the first atlas of aortic cells [3]. This report established a resource recording the gene expression features of ∼1000 aortic cells, which were mainly ECs and fibroblasts.

The aorta is shaped like a candy cane; it can be divided into ascending, arch, thoracic and abdominal segments. Their different anatomical structures give rise to various hemodynamic microenvironments inside the aortic tubes. To understand how each type of aortic cell responds to the subtly changing biomechanical microenvironment, and how they cooperatively regulate homeostasis of aortic function, the spatial heterogeneity of aortic cells deserves deep investigation and surveys of the RNA profiles of individual cells from different aortic segments would help to address these questions [4].

Lifestyle characteristics such as a high salt intake, obesity and abnormal blood glucose, lead to metabolic syndrome that includes adverse effects on the aorta by disturbing the homeostasis of ECs, fibroblasts, adipocytes and immune cells [5,6]. Investigation of pathogenic changes in cell composition and function provides promising targets for preventing and treating aortic diseases. Previously, Cochain *et al.* and Winkels *et al.* used scRNA-Seq to reveal a heterogeneous population of immune cells in mouse aorta and discovered several atherosclerosis-associated immune cell types under a western-type diet [7,8]. Enlightened by their reports, we believe it is important to further characterize the spatial heterogeneity of aortic cells across segments under conditions of high blood glucose levels, or high dietary salt or high fat intake.

Here, we used scRNA-Seq to analyze (i) control mouse aorta and its four anatomical segments; and (ii) the aorta and its four segments from mice fed a high-salt or high-fat diet, or from mice with high plasma glucose. We revealed heterogeneity within a certain cell type, between different aortic structures and in the presence of different cardiovascular risk factors. These data provide a better understanding of altered aortic cellular composition associated with biomechanical and biochemical changes in the vascular system.

## RESULTS

### Single-cell survey of mouse aorta

#### Overview of aortic cells

**Figure 1.**
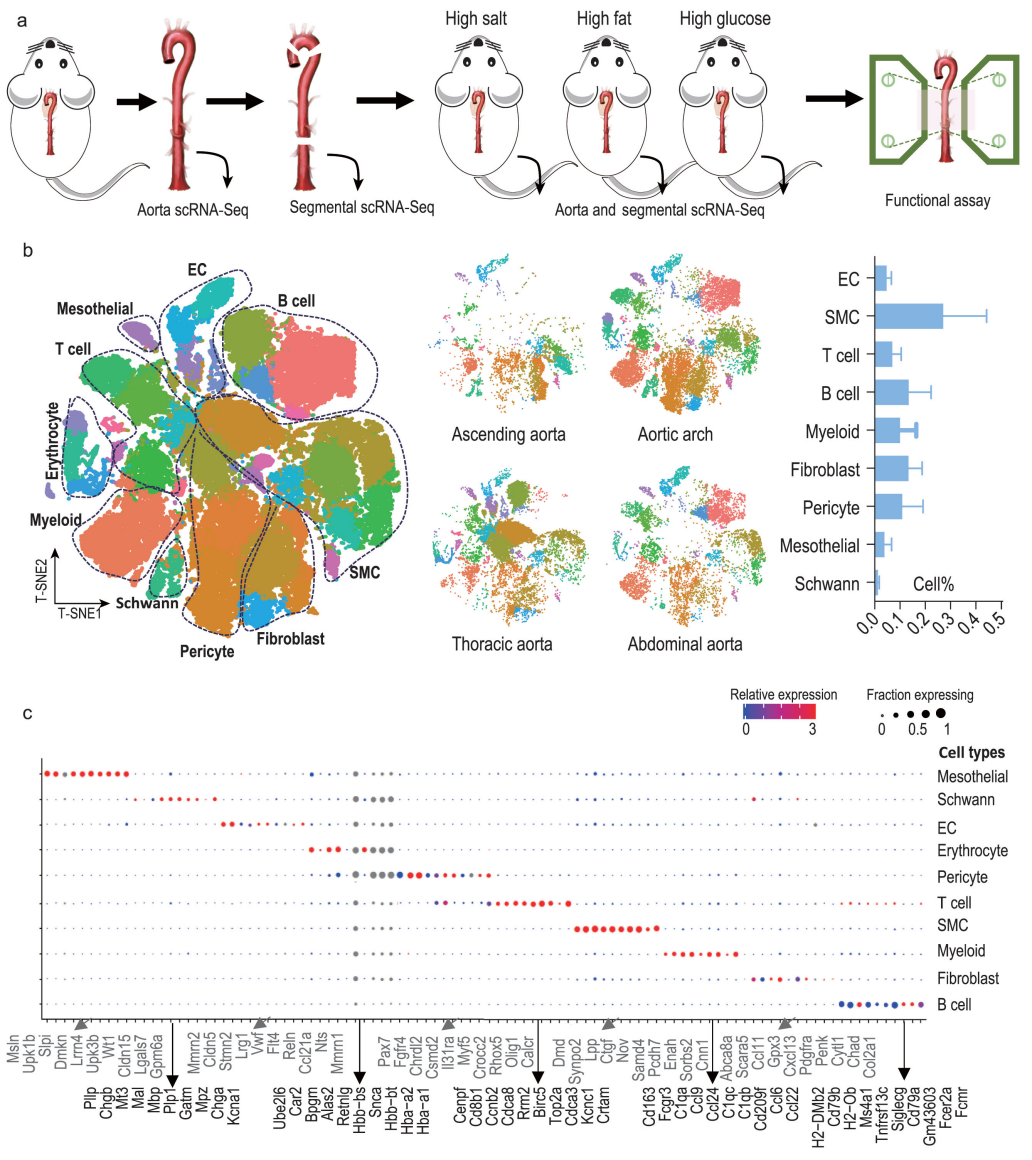
Global and segmental analysis of expression profiles of single cells from mouse aorta. (a) Overview of the experimental design. (b) T-distributed stochastic neighbor embedding (t-SNE) visualizing single cells from intact aorta (*n* = 4 aortas), the ascending segment (*n* = 3 aortas), aortic arch (*n* = 3 aortas), thoracic segment (*n* = 3 aortas) and abdominal segment (*n* = 3 aortas). Left, t-SNE of all cells from intact aortas and segments. Middle, t-SNE of cells from aortic segments. Right, bar graph showing the proportions of cell types in aorta, where erythrocytes are not included as their percentage depends on the intracardiac perfusion processes. (c) Dot plots of 10 cell-type signatures. Dot size, proportion of cells; colors, expression levels.

In this study, we used scRNA-Seq to profile a total of 216 612 single cells from healthy mouse aortas, four segments of healthy aortas, as well as aortas and aortic segments from mice fed a high-salt or high-fat diet, or with high plasma glucose (see Fig. 1a and Table S1 for the numbers of experimental replications and profiled cells). Overall, the sequencing generated a median gene value of 1786, with 118 608 confidently-mapped reads per cell and a 67.2% mean transcriptome mapping rate per cell. The median number of unique molecular identifiers (UMIs) was 6272. The average proportion of transcript counts derived from mitochondria-encoded genes was 7.6%.

Single cells from four healthy aortas were clustered using graph-based clustering followed by Louvain modularity optimization. The clusters were projected onto t-distributed stochastic neighbor embedding (t-SNE) plots and segregated into 35 distinct cell clusters (Fig. 1b). Based on the expression values of known marker genes [log_2_ fold-change (log_2_FC) > 1.5, *P* < 0.05 *versus* other clusters] [4,7,8], we identified 10 major cell types: ECs (*PECAM1* and *CDH5*) [9,10], vascular smooth muscle cells (SMCs; *CNN1*, *ACTA2* and *MYH11*) [11,12], T lymphocytes (*PTPRC* and *CD3E*), B lymphocytes (*BANK1* and *H2-DMb2*) [13], myeloid cells (*CD68* and *CD163)* [14,15], fibroblasts (*PDGFRA* and *VIM*) [16–19], pericytes (*PDGFRB* and *CSPG4*) [20], mesothelial cells (*LRRN4*, *UPK3B* and *MSLN*) [21,22], Schwann cells (*PLP1* and *CNP*) [23,24], and erythrocytes (*HBB-BT*, *HBA-A1* and *HBA-A2*) (Figs 1b and S1a). Quantitatively, SMCs, fibroblasts and pericytes were the largest components (Fig. 1b). A list of signature genes was identified for each major cell population (Table S2), and the expression patterns of the top 10 signature genes are shown in Fig. 1c.

#### Spatial diversity of cell types across aortic segments

To reveal differences in the cellular composition between the four aortic segments, we separately profiled single cells from the ascending aorta, aortic arch and thoracic and abdominal aortas in independent experiments (see strategy in Fig. 1a). Sequencing data showed that the 10 major cell types were identified in all segments (Fig. 1b). However, the percentage of each cell type differed across segments (Fig. S1b), where no major batch effect was found because all replicates contributed to all clusters (Fig. S1c). The aortic arch showed the most distinct features of cell composition. The curved structure of the arch is athero-susceptible. As expected [25], more immune cells were present within the healthy aorta arch than other segments, especially macrophages, as validated by CD68 immunostaining (Fig. S1b and d). A prominent proportion of Schwann cells was found in the aortic arch as indicated by PLP1 immunostaining (Fig. S1b and d) [26], and this finding is consistent with previous reports that Schwann cells are more frequently located in the aortic arch to regulate heart rate and blood pressure [27]. Pericytes were first found in capillaries; they regulate a variety of important vascular functions from vascular remodeling to the generation of atherosclerosis [28]. Accumulating evidence indicates that pericytes are also present in large arteries, including the aorta [28]. Here, we found that the aortic arch contained more pericytes than the other segments (Fig. S1b and d), suggesting that this segment requires the ‘pleiotropic’ pericytes to help maintain homeostasis of the microenvironment [28]. Together, these results confirm cellular heterogeneity across aortic segments.

#### Characterizing subsets of aortic cells and their spatial heterogeneity

**Figure 2.**
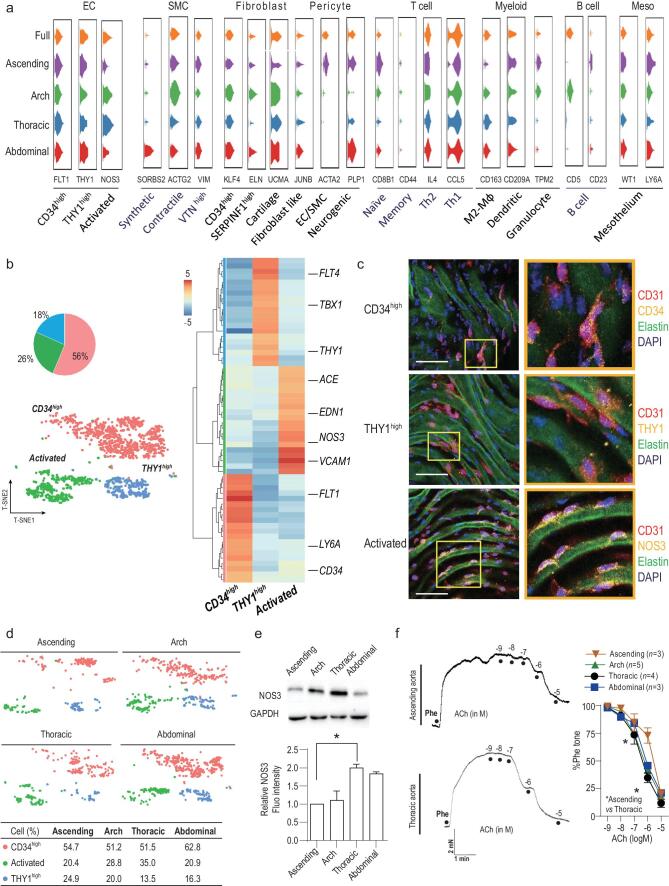
Characterizing aortic ECs and their spatial distribution. (a) Violin plots showing distribution of expression of selected marker genes across cell subpopulations. (b) t-SNE of 1663 ECs (collected from eight mice). Pie graph represents the proportions of EC subpopulations. Heatmap shows 20 gene signatures of the subpopulations. (c) EC subpopulations analyzed with immunofluorescence. Three subpopulations were labeled with respective markers: CD34 for contractile *CD34*^high^; THY1 for synthetic *THY1*^high^; NOS3 for activated EC. (d) t-SNE demonstrating the regional distribution of EC subpopulations in the ascending aorta, aortic arch, thoracic aorta and abdominal aorta. The percentages of each cell subpopulation are summarized in the table. (e) Western blots comparing NOS3 expression among the four aortic segments. (f) Original traces and summary of activated EC-related vasodilation recorded by myography. Scale bars, 50 μm. *P < 0.05, one-way ANOVA in (e); two-way ANOVA in (f).

To reveal the heterogeneity within a specific aortic cell type, and characterize the spatial diversity of sub-clusters across an aortic segment, we first sub-clustered the major aortic cell types according to their gene expression profiles. To do this, we used t-SNE followed by graph-based or k-means-based clustering. Second, we recorded the aortic locations of each identified subpopulation. The overall results are summarized in Fig. 2a in violin plots, which illustrate the expression patterns of different marker genes for subpopulations and their regional locations. The characteristic of each aortic cell type is described below.

#### Subpopulations of aortic ECs and their spatial heterogeneity

We identified three populations of ECs by their expression profiles and presence among segments (Fig. 2b). Cells designated as *CD34*^high^ ECs accounted for ∼56% of all ECs. They strongly expressed *CD34* and *LY6A* (log_2_FC > 3 *versus* the other subpopulations, *P* < 0.001). The other *THY1*^high^ EC subpopulation (∼18% of all ECs) strongly expressed *THY1*, *TBX1* and *FLT4* (log_2_FC > 3, *P* < 0.001) (Fig. 2b). We validated the *CD34*^high^ and *THY1*^high^ subpopulations by immunostaining for CD34 and THY1 in ECs (Fig. 2c). GO analysis of the highly expressed genes in *CD34*^high^ ECs (465 genes with log_2_FC > 1.5 *versus* the other subpopulations, *P* < 0.05) and in *THY1*^high^ ECs (610 genes with log_2_FC > 1.5 *versus* the other subpopulations, *P* < 0.05) showed enrichment in pathways that are modulated by CD34, THY1 and LY6A, including those involved in endothelial proliferation and vascular system development [29,30] (Table S3). Our further analysis below (see Fig. 4b) also supported the idea that these *CD34*^high^ and *THY1*^high^ ECs respond to paracrine signals to proliferate and promote vasculogenesis. For example, *FLT1* and *KDR* (major vascular growth factor receptors) were upregulated in *CD34*^high^ ECs (log_2_FC = 2.5 and 1.8 respectively *versus* the other subpopulations, *P* < 0.001), strongly suggesting that this subpopulation plays a role in supporting vasculogenesis. However, the functions and differences between the *CD34*^high^ and *THY1*^high^ subpopulations require further exploration. CD34 is one of the markers for EC progenitor cells [16,31], but the proportion of *CD34*^high^ was too high to be EC progenitor cells (some may be, but they were not identified here). Interestingly, a recent work [32] may help to further understand the features of *CD34*^high^ ECs. Lukowski *et al.* explored the single-cell gene expression profiles of CD34-sorted aortic ECs. The cell-sorting method enabled collection of rare CD34-postive cell subtypes. Their study then identified both EC progenitors and differentiated ECs within these CD34-positive aortic ECs, with distinct but intercommunicated functions.

Unlike the *CD34*^high^ and *THY1*^high^ subpopulations, GO analysis of the 416 significantly changed genes (log_2_FC > 1.5 *versus* the other subpopulations, *P* < 0.05) in the last subpopulation of ECs was enriched in pathways associated with vessel dilation and blood pressure regulation. Consistently, they strongly expressed genes (log_2_FC > 3 *versus* the other subpopulations, *P* < 0.001) that are known to regulate vascular tone, such as *NOS3* (validated in Fig. 2c), *EDN1* and *ACE*, and canonical EC markers (*VCAM1*) (Fig. 2b)*.* Therefore, we named these cells ‘activated’ ECs.

We then analyzed the regional diversity of the three EC subpopulations and found that all ECs subpopulations were distributed throughout the aorta, but with differing percentages (Fig. 2d), which was also validated with immunostaining with marker proteins of the EC subpopulations including NOS3 (activated), CD34 (CD34^high^) and THY1 (THY1^high^) (Fig. S2a). Because the activated ECs may regulate vascular tone that can be directly measured, we then assessed the spatial heterogeneity of activated EC-related differences in vascular segments. The frequency of activated ECs was higher in the thoracic segment than in the other three segments (Figs 2d and S2a), and the results were also supported by western blots for NOS3 protein (Fig. 2e). On the contrary, the ascending aorta contained the lowest proportion of activated ECs (Figs 2d, e and S2a). We then compared the ACh-induced relaxation across the four segments to measure differences in EC-dependent vasodilation. Wire myography supported the scRNA-Seq analysis, in which the thoracic segment showed stronger EC-dependent vasodilation, while the response was weaker in the ascending aorta (Fig. 2f). Therefore, these results suggest that the spatial heterogeneity of activated ECs may contribute to different regulation of vasodilation across aortic segments.

Previously, Kalluri *et al.* [33] analyzed aortic EC subtypes using scRNA-Seq, and they identified two blood vessel EC subtypes (*VCAM* and *CD36*-expressing, respectively). Here, we analyzed the gene expression correlation of these two EC subtypes with the three EC subtypes identified in our study. Interestingly, we found that *VCAM1*-expressing ECs showed great similarity in gene expression profiles of activated ECs; further, *CD36*-expressing are highly similar with *CD34*^high^ ECs defined in our study (Fig. S2b). Furthermore, both studies indicated a higher proportion of activated ECs (or *VCAM1*-expressing ECs) in aortic segments with less curvature, such as thoracic segment. Therefore, our study on EC subtypes is consistent with that of Kalluri *et al.*, where they also identified similar aortic cell composition as we show in Fig. 1b. Of note, the subsequent data analysis and functional validation from Kalluri *et al.* and our study are complementary. Kalluri *et al.* focused on the extracellular matrix production, lipid handling and angiogenesis pathways, while we identified spatial heterogeneity of vascular tone regulation.

#### Subpopulations of aortic SMCs and their spatial heterogeneity

**Figure 3.**
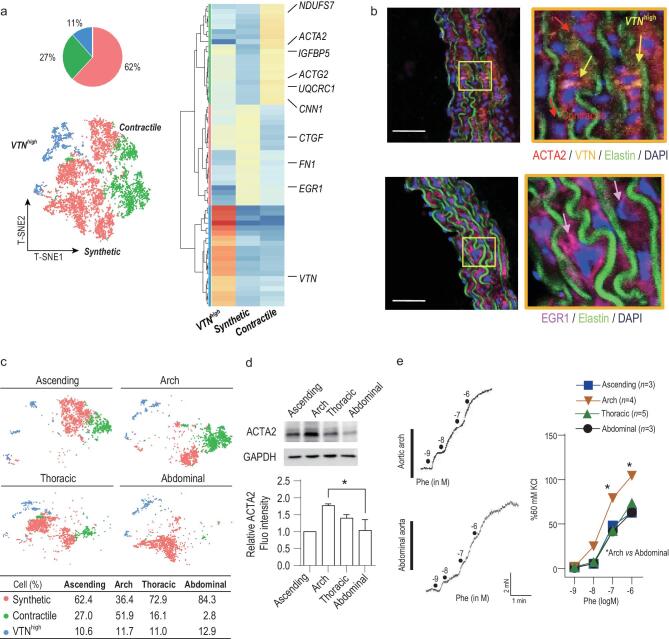
Characterizing aortic SMCs and their spatial distribution. (a) t-SNE of 7806 SMCs (collected from eight mice). Pie graph represents the proportions of SMC subpopulations. Heatmap shows 20 gene signatures of the subpopulations. (b) SMC subpopulations analyzed with immunofluorescence. Three subpopulations were labeled with respective markers: ACTA2 for contractile SMC; EGR1 for synthetic SMC; VTN for *VTN^high^* SMC. (c) t-SNE demonstrating the regional distribution of SMC subpopulations in the ascending aorta, aortic arch, descending aorta and thoracic aorta. The percentages of each cell subpopulation are summarized in the table. (d) Western blots of ACTA2 expression in the four aortic segments. (e) Original traces and summary of contractile SMC-related constriction recorded by myography. Scale bars: 50 μm. *P < 0.05, one-way ANOVA in (d); two-way ANOVA in (e).

We annotated SMCs into synthetic, contractile and *VTN^high^* based on their expression profiles and presence among segments (Figs 2a and 3a). The presence of synthetic and contractile subpopulations is consistent with previous reports [34]. Synthetic SMCs accounted for the highest proportion (∼62% of all SMCs) and expressed genes associated with proliferation and migration (*EGR1*, *FN1* and *CTGF*, log_2_FC > 3, *P* < 0.001) (Fig. 3a); their potential functions were also revealed in GO analysis for 140 significantly changed genes (Table S3). Contractile SMCs (∼27% of all SMCs) encoded signals that are thought of as ‘contractile’, including *ACTG2*, *ACTA2*, *CNN1* and *TAGLN* (log_2_FC > 3, *P* < 0.001) (Fig. 3a). They also expressed genes (*UQCRC1* and *NDUFS7*, log_2_FC > 2, *P* < 0.001) enriched in biological processes (GO analysis in Table S3) implicated in aerobic respiration and oxidation-reduction to meet the energy requirements of contraction [35].

Similar to contractile SMCs, *VTN^high^* cells expressed *ACTG2* and *ACTA2* (Fig. 3a), suggesting they can contract. They also strongly expressed *VTN* (*VTN^high^*, log_2_FC = 3.5, *P* < 0.001), which encodes a glycoprotein that was first detected in serum and plasma. It is synthesized in the liver [36], but can also be produced by SMCs in carotid arteries [37,38]. Here, the scRNA-Seq results showed the presence of a *VTN*-expressing subpopulation of aortic SMCs that accounted for ∼11% of all aortic SMCs. Their function in aorta deserves further study. They expressed the contraction-related marker genes *ACTG2* and *ACTA2*, and the motility-associated genes *VTN* and *IGFBP5* (log_2_FC > 3, *P* < 0.001 *versus* other SMCs), consistent with previous reports that *VTN*-expressing SMCs modulate SMC migration and contractility [37,38]. We then performed immunostaining to validate the presence of synthetic, contractile and *VTN^high^* subpopulations (Fig. 3b).

Next, we analyzed the heterogeneity of the spatial organization of SMCs, and validated with immunostaining for marker proteins in the three subpopulation including EGR1 (synthetic) and ACTA2 (contractile) and VTN (*VTN^high^*) (Fig. S3a). There was a marked difference in composition of contractile and synthetic SMCs across aortic segments, but less in *VTN^high^* SMCs (Figs 3c and S3a). Contractile SMCs account for 52% of total aortic cells in the aortic arch, but only 2% in the abdominal segment, which contained the greatest proportion of synthetic SMCs (Fig. 3c). The spatial diversity of contractile SMC composition was also validated with western blots (Fig. 3d). Next, we found that the spatial diversity of contractile SMCs was tightly associated with functional differences. Using wire myography to measure phenylephrine-induced (an α-1 adrenergic agonist) smooth muscle contraction, we found that the contractile power of the aortic arch was significantly greater in response to phenylephrine, while the abdominal aorta showed a weaker response (Fig. 3e), suggesting that differences in contractile SMC composition may contribute to different contractile power across segments.

Of note, SMCs assigned to the synthetic phenotype segregated in the t-SNE coordinates according to the aortic segment from which they came (Fig. 3c). Synthetic SMCs from the ascending aorta exhibited molecular features similar to those from the aortic arch (Spearman *r* = 0.655, *P* = 0.0003, Fig. S3b); both highly expressed genes associated with SMC proliferation, such as PDE1C, a major regulator of smooth muscle proliferation [39] (Log_2_FC > 1.5, *P* < 0.05) (Fig. S3c). These findings are consistent with the function of synthetic SMCs and also indicate the importance of SMC proliferation in the ascending and arch segments. Further, gene expression of synthetic SMCs in the thoracic and abdominal segments was less correlated with the other segments. *BMP10* in the thoracic aorta, and *KIT* and *RGS5* in the abdominal aorta, ranked as the top genes compared to other segments (Log_2_FC > 1.5, *P* < 0.05) (Fig. S3c). These genes have implications for SMC proliferation and development [40–43]. Their spatial expression in the thoracic and abdominal segments, but not in the aortic arch and ascending segment, may inspire further studies on their physiological roles in hemodynamic responses.

#### Subpopulations of aortic stromal cells and their spatial heterogeneity

Stromal cells include fibroblasts and pericytes. Based on their gene expression profiles, we identified six subpopulations (Figs 2a and S4a).

Among the aortic fibroblasts, we first identified *CD34^high^* fibroblast cells that strongly expressed *CD34* (log_2_FC = 3.2, *P* < 0.001 *versus* other stromal cells) (Fig. S4a), and this was validated with immunostaining (Fig. S4b)*. CD34* has been reported to be a marker gene of fibrocytes. However, fibrocytes also express a combination of marker genes including *CD45* and smooth muscle actins, whose expression changes when fibrocytes move from bone marrow to tissues [44]. Given the critical roles of fibrocytes in other cardiovascular tissue, such as the pulmonary artery and heart [1,44], it will be important to determine whether the *CD34^high^* fibroblasts act as aortic fibrocytes and mediate functions like tissue repair, fibrosis, contraction and angiogenesis in future work [44]. Here, our scRNA-Seq results provided clues for such studies: we found that the 236 significantly changed genes in *CD34^high^* fibroblasts (log_2_FC > 1.5, *P* < 0.05 *versus* other stromal cells) were enriched in GO biological processes regulating SMC hypertrophy (*IGFBP5*, *PI16* and *HN1*), angiogenesis (*ANXA1*, *KLF4* and *TGFBR2*) and extracellular matrix organization (*FN1*, *LAMC1*, *TNXB*, *ADAMTS5*, *TIMP2*, *FBN1* and *COL14A1*) (Table S3), suggesting a multifunctional role of *CD34^high^* fibroblasts.

Another fibroblast population was distinguished as *SERPINF1^high^* fibroblasts highly expressed *SERPINF1* (log_2_FC = 3.2, *P* < 0.001; Figs 2a and S4a; see Table S3 for potential functions from GO analysis). *SERPINF1* encodes a glycoprotein with anti-oxidant, anti-angiogenic, anti-thrombotic, anti-atherosclerotic and anti-tumorigenic properties [45,46]. Many studies have elucidated its important pathophysiologic role in the cardiovascular system [46]. Our results support the previous finding that *SERPINF1* is expressed in cardiovascular fibroblasts [47], but its function in the aorta requires further investigation.

The third fibroblast subpopulation consisted of cartilage-like fibroblasts that expressed genes associated with cartilage development: *OTOR* (see Fig. S4b for the validation of this gene expression), *ACAN*, *UCMA* and *COMP* (Log_2_FC>5, *P *< 0.01 *versus* other stromal cells) (Fig. S4a). They also expressed the rare vascular collagens *COL2A1*, *COL9A1–3* and *COL11A2* (log_2_FC > 1.5, *P* < 0.05 *versus* other stromal cells). This is the first identification of fibroblasts that strongly express cartilage-related genes in a blood vessel. Referring to previous reports on fibrocartilage, these cells might modulate the tensile strength and vascular resilience of the aorta [2].

When examining the regional distribution of the fibroblast subpopulations, we found that both *CD34^high^* and cartilage-like fibroblasts were preferentially distributed in the aortic arch (see Fig. S4b for immunostaining validation; Fig. S4c for t-SNE plot). *SERPINF1*^high^ cells did not show spatial differences. Considering the potential function of *CD34^high^* and cartilage-like fibroblasts, their spatial diversity again suggests that the cellular and vascular architecture of the aortic arch differs from that in other segments.

Another population of stromal cells were pericytes. These cells did not show clusters clearly separate from fibroblasts in the t-SNE plot (Fig. S4a), suggesting that they share similar gene expression profiles. However, they were identified as pericytes because they expressed *PDGFRB* and *CSPG4* (log_2_FC > 1.5, *P* < 0.05 *versus* fibroblasts) (Fig. S4b). Based on differences in expression profiles, pericytes fell into three subpopulations (Figs 2a and S4a, see Table S3 for potential functions from GO analysis). Fibroblast-like pericytes co-expressed the fibroblast marker *PDGFRA* (log_2_FC = 2.3, *P* < 0.001) and high levels of genes associated with cell proliferation (*FGFR4*, *SFRP1*, *JUNB* and *IGF*, log_2_FC > 1.5, *P* < 0.05 *versus* other stromal cells). They were mainly distributed near the aortic arch as indicated by both scRNA-Seq and immunostaining (Figs S4b and S4c). Second, EC/SMC pericytes expressed genes associated with extracellular matrix construction (*CRISPLD2*, *CYR61*, *MFAP4*, *COL4A1* and *MYH11*) (log_2_FC > 1.5, *P* < 0.05 *versus* other stromal cells), but these genes were weakly expressed in other pericyte and fibroblast subpopulations. These cells also had features resembling both ECs and SMCs, expressing the marker genes *PECAM1* and *ATCA2*, suggesting myogenic and endotheliogenic properties. Notably, EC/SMC pericytes were prominently located in the ascending aorta as shown in the t-SNE plot (Fig. S4c). We then validated the expression of one of the marker genes, *PECAM1*, in EC/SMC pericytes with immunostaining, and showed that these *PECAM1*-expressing pericytes were distributed in the ascending aorta (Fig. S4b). Finally, neurogenic pericytes were enriched in Schwann cell markers (*PLP1* and *CNP*, log_2_FC = 5.6 and 4.6 respectively, *P* < 0.001). Like Schwann cells, these pericytes were located near the aortic arch.

#### Subpopulations of aortic immune cells and their spatial heterogeneity

We then characterized the populations of aortic immune cells, and most of our results were consistent with previous studies [7,8] Both B1 (*CD27*, *CD5* and *CD11B*) and B2 B cells (*CD21*, *CD22* and *CD23*) were identified (Figs 2a and S5a). Further, a total of four clusters of T cells emerged (Figs 2a and S5b): a combination of naïve T cells (*LEF1*, *SELL* and *CCR7*) and Tregs (*FOXP3*); memory T cells (*CD44*); Th2 cells (*IL4*); and Th1 cells (*IFNG* and *TBX21*) [48]. Moreover, the myeloid population was divided into three clusters (Figs 2a and S5c): M2-macropahges (*MRC1* and *CD163*), monocyte-derived dendritic cells (*CD209A*, *CD74* and *FLT3*) and granulocytes (*S100A8*, *S100A9* and *NGP*).

When the immune cells of the four aortic segments were separately examined, we found that B2 cells, naïve T/Tregs and M2-macrophages were all strongly localized in the aortic arch and abdominal segments (Fig. S5a–c). Given the ‘protective’ or ‘repair’ feature of these immune cells [8,49], the results indicate that the aortic arch and abdominal segments are areas susceptible to vascular damage.

#### Characterizing subsets of mesothelial cells and their spatial heterogeneity

Mesothelial cells are a source of endothelium, SMCs and cardiomyocytes [50–52]. We identified progenitor-like mesothelial cells in the aorta. One subpopulation comprised *WT1^high^ CD34^high^* cells (Fig. S6). Another was the *LY6A^high^* subpopulation that was rarely found in the abdominal segment; this subpopulation also expressed high levels of SMC-specific genes (*ACTA2*, *TAGLN* and *CNN1*, log_2_FC = 1.52, 2.02 and 1.64 respectively, *P* < 0.05 *versusWT1^high^ CD34^high^*), suggesting that they play a role in the mesothelial-SMC transition [51].

#### Intercellular network of aortic cells

**Figure 4.**
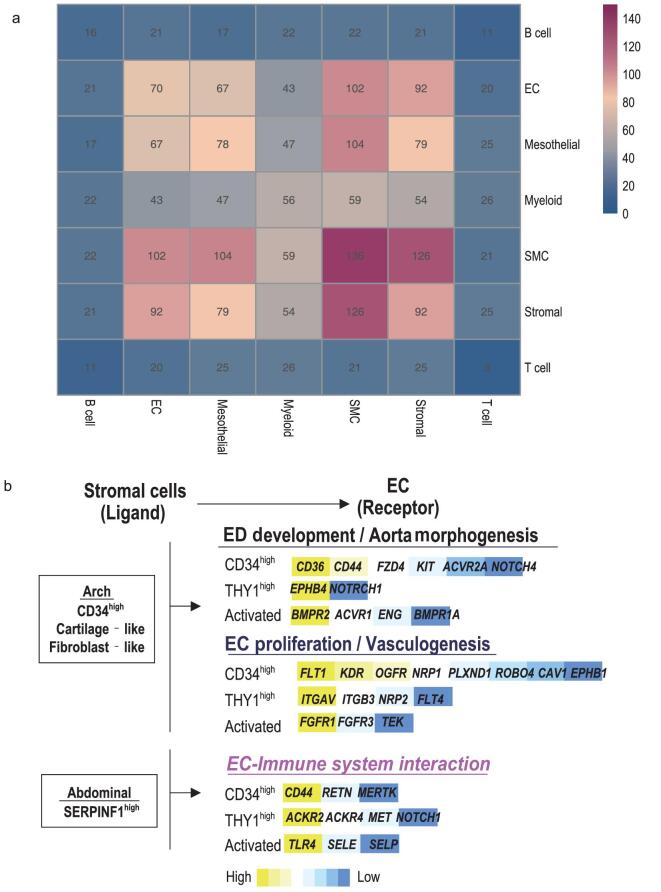
Intercellular networks. (a) Putative ligand and receptor-based cell–cell interaction between aortic cells. High value means strong cell–cell interaction. (b) EC–stromal cell communication constructed when ligands from stromal cells and reciprocal receptors on ECs were both highly expressed. The names and relative expression of EC receptors are listed.

We constructed intercellular networks to reveal the cellular landscape of the different aortic cells using CellPhone DB[53]. The cell–cell interactions were calculated by putative signaling between differentially expressed receptors and their differentially released ligands from aortic cell types identified in this study. In general, most of the subpopulations formed interconnected networks. The stromal cells *versus* ECs and SMCs as well as ECs *versus* SMCs showed strong correlations (Fig. 4a). Other notable interaction included mesothelial cells *versus* SMCs, ECs and stromal cells, suggesting that they have interconnected physiological functions, which deserve further investigation. Such interactions were also seen between different subpopulations of stromal cells, ECs, SMCs and mesothelial cells, or within subtypes of SMCs or stromal cells (Fig. S7a).

We then analyzed the spatial differences in EC–stromal cell interactions in detail, based on three arguments: (i) both ECs and stromal cells were differentially distributed between the aortic arch and descending segments (Figs S1b and 2a), suggesting that they are regulated by vascular biomechanical changes; (ii) these changes in microenvironments finally determine vascular functions which are EC-dependent [54]; and (iii) there is a tight connection between stromal cells and ECs [55,56] (Fig. 4a). We assessed the heterogeneity of stromal cell ligands *versus* EC receptor communications using an online tool for analyzing ligand–receptor interactions [57], and compared these interactions between the curved aortic arch and the straight abdominal aorta (Figs 4b and S7b). To show how the signal produced by fibroblast ligands affects activity of ECs, upregulated receptors in ECs (log_2_FC > 1.5, *P* < 0.01) were listed together with their enriched biological processes in GO (examples in Fig. 4b and details in Fig. S7b). First, the aortic arch was populated with *CD34^high^* fibroblasts, cartilage-like fibroblasts, neurogenic pericytes and fibroblast-like pericytes. The expressed ligands bind to corresponding receptors that were highly expressed in three types of ECs. GO analysis of these receptors showed that they evoke functions including EC differentiation and aorta development. These ligands also evidently support EC survival in all three EC subpopulations *via* activated EC proliferation, migration and vasculogenesis pathways. For example, *PDGFC* (log_2_FC = 3.5), *PI*GF (log_2_FC = 2.2) and *FIGF* (log_2_FC = 1.5) were genes of ligands elevated in cartilage-like fibroblasts, neurogenic pericytes and *CD34^high^* fibroblasts, respectively. They encodes ligands that all target their receptors in *CD34*^high^ ECs *FLT1* (log_2_FC = 2.5) to promote endothelial proliferation [58]. These findings are consistent with former arguments that regions of flow disturbance, like the aortic arch, have EC transcript profiles with proliferative features, which may provide sustained renewal of the EC population in the demanding microenvironment of the aortic arch [59–62].

Second, stromal cells in the thoracic and abdominal segments were less diverse; these regions were populated with more *SERPINF1^high^* fibroblasts. Interestingly, the ligands of *SERPINF1^high^* fibroblasts evoke EC receptors that are associated with EC-immune system interactions. For example, fibroblast *SERPING1* (log_2_FC = 1.8) targets activated EC *ECLE* (log_2_FC = 4.7), and the latter stimulates vascular inflammation [63]. Therefore, ECs in the thoracic and abdominal segments are more likely to undertake the task of communicating with the immune system.

### Single-cell survey of at-risk aortic cells

#### Overview

**Figure 5.**
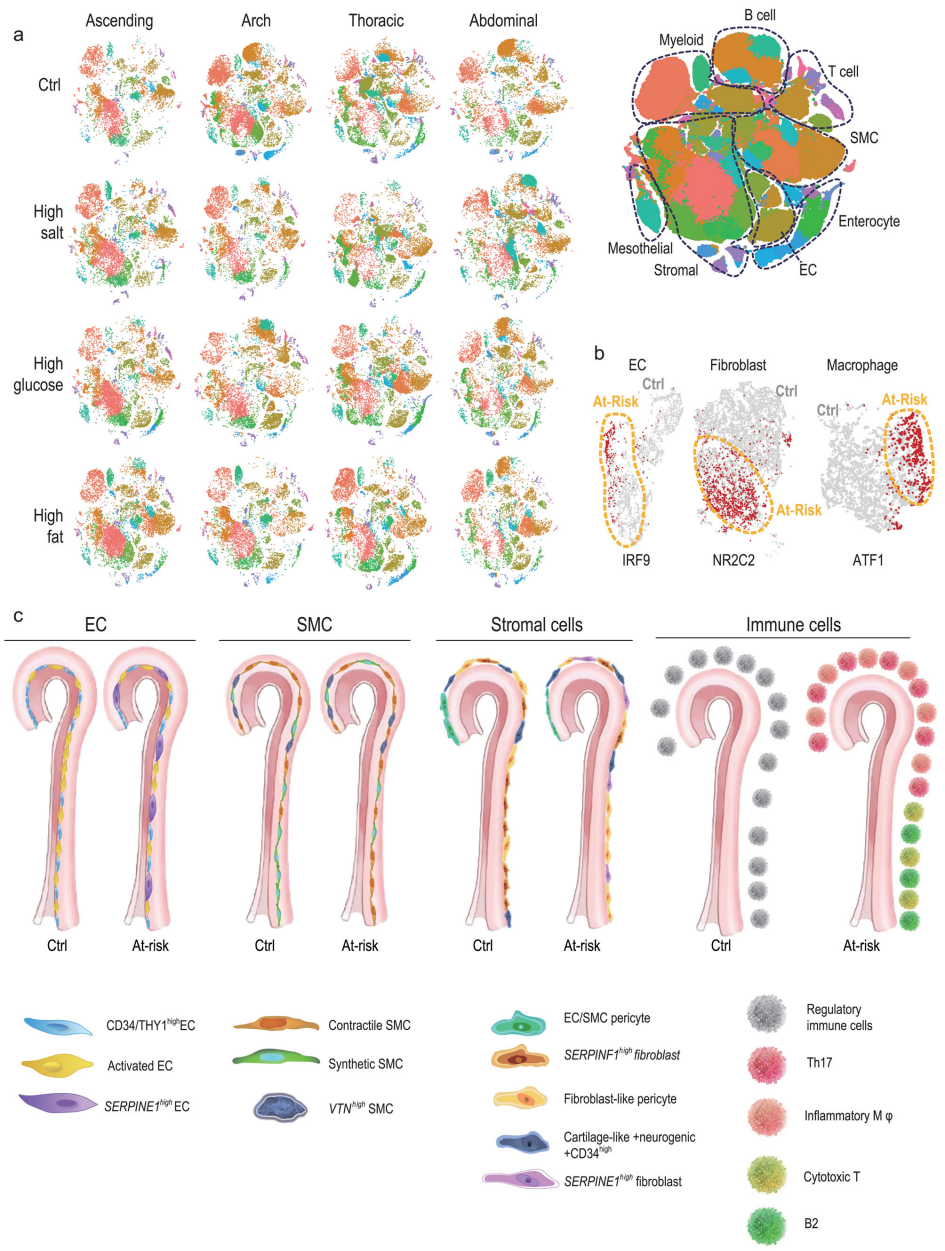
Aortic cell responses to high-glucose, high-salt and high-fat conditions. (a) Overview of cell distribution (shown in t-SNE) in the ascending, arch, thoracic and abdominal segments from control mice and those with high-salt intake, high-fat intake and high plasma glucose levels (*n* = 2/segment for each condition). A t-SNE plot combining all of the cells is shown in the right panel with cell type annotation. (b) t-SNE plot displaying three regulons in ECs, fibroblasts, and macrophages of the abdominal segment (details in Methods). Red dots, single cells with active indicated regulons; orange-circled area, single cells from at-risk aorta; others, single cells from healthy aorta. (c) Summary cartoon displaying cell composition changes across segments, and between control aorta and common features of aortas from mice with high-salt intake, high-fat intake and high plasma glucose levels.

Another major goal of our study was to uncover the features of aortic cellular responses to three commonly recognized risk factors for vascular disease [64–67]. We therefore profiled aortic cells from mice exposed to a high-salt diet, a high-fat diet and high plasma glucose (Figs 1a, 5a and S8). To do this, whole aortas and aortic segments were obtained from these at-risk mice and RNA of single cells were sequenced.

Here, we mainly explored the common features of aortic cells under three at-risk conditions. However, at-risk aortas also showed specific responses to each treatment, which deserves further investigation with our scRNA-Seq data in future. From the aspect of cellular and spatial heterogeneity, the cellular composition shifted in the three types of at-risk aorta and in all segments (Fig. 5a; Table S4). To summarize, fibroblasts and pericytes increased substantially in at-risk aortas, most markedly in the thoracic and abdominal segments. Myeloid and T cells also increased prominently by ∼25% and 20%, respectively, and the at-risk aortic arch and abdominal segment both showed evident changes. The tendency for change in immune cells was consistent with that in atherosclerotic mice [7,8]. The above changes of aortic cell composition across segments were validated with immunostaining in all three types of at-risk aorta (Fig. S9).

The gene expression profiles of ECs, stromal cells and immune cells were analyzed before and after exposure to vascular risk factors. GO analysis of highly dysregulated genes (log_2_FC > 1.5, *P* < 0.05) indicated that these factors compromised the proliferative ability of ECs, destroyed the matrix organization by affecting fibroblasts and pericytes, and activated the inflammatory response of immune cells (Fig. S10). We also identified genes that commonly changed in the indicated cell types from aortas from mice with a high-salt diet, high-fat diet and high plasma glucose (indicated in the heatmap of Fig. S10). For example, in pericytes, 36 genes were significantly decreased in at-risk aortas (log_2_FC > 1.5, *P* < 0.05); these may subsequently interfere with the formation of extracellular matrix by diminishing the expression of *Col10a1*, *Col11a2*, *Col2a1*, *Col9a1*, *Col9a2*, *Col9a3*, *Mia* and *Acan*. Also, we identified lists of downregulated genes in at-risk ECs, SMCs and fibroblasts, and upregulated genes in immune cells. However, their specific functions in these cell types and the implication for cardiovascular disease are unknown, serving as new targets for future studies.

#### Characterizing subsets of aortic cells under a high-salt diet, a high-fat diet and high plasma glucose conditions

We next investigated changes in the aortic cell subpopulations with a high-salt diet, a high-fat diet or high plasma glucose. One of the significant changes in the EC population was the emergence of ECs with *OCLN*^low^*SERPINE1*^high^ characteristics (Fig. S11a). *OCLN* is associated with the tight junctions of ECs while *SERPINE1* mediates the degradation of extracellular matrix and is pro-atherogenic [68]. These cells were dispersed throughout the aorta under all three risk conditions. Indeed, aortas from at-risk mice showed a marked increase in permeability (Fig. S11b). Given the spatial distribution and physiological function of *OCLN* and *SERPINE1*, it is worth exploring how strongly *OCLN*^low^*SERPINE1*^high^ ECs contribute to aortic permeability, and their mechanisms in pathological processes. Furthermore, the scRNA-Seq data indicated that composition of activated ECs decreased in all at-risk groups and all segments compared to healthy aortas (Fig. S12 compared to Fig. 2d), and myography results also indicated that ACh-induced vasodilation was largely decreased (Fig. S12 compared to Fig. 2f). For example, the high composition of activated ECs in thoracic segments in control aorta decreased dramatically in all at-risk groups (from 35±1.3% to lower than 20% in all three at-risk groups). Consistently, the EC-related relaxation significantly decrease (Fig. S12). These results may again indicate the role of activated ECs in aortic dilation.

Subpopulations of SMCs also showed extensive changes (Fig. S13). *VTN^high^* SMCs decreased extensively in all three groups. Composition of contractile SMCs increased in all at-risk groups and all segments compared to healthy aortas (Fig. S14 compared to Fig. 3c), and myography results also indicated that the contractile power of at-risk aorta increased (Fig. S14 compared to Fig. 3e). For example, synthetic SMCs dominated the abdominal segment in healthy mice, but under all three at-risk conditions the proportion of contractile SMCs in this segment increased from 2.8 ± 1.3% to 37.3 ± 8.9% of all SMCs, accompanied with significantly increased contractile power (Fig. S14).

Fibroblast heterogeneity increased under the risk conditions (Fig. S15a). Some adipogenic fibroblasts expressing both fibroblast and adipocyte markers (*GPD1*, *ADIPOR2* and *ADIPOQ*) were identified in the aortas from mice with high blood glucose, suggesting a tendency to accumulate adipocytes. Further, some fibroblasts highly expressed the vascular disease-related *SERPINE1* [68] (also seen in *OCLN*^low^*SERPINE1*^high^ ECs), and they were mainly found in the high-fat aortic arch. Notably, fibroblast-like pericytes showed the most extensive changes among all three at-risk models. They were located in the aortic arch in healthy mice, but mainly in the abdominal segment in all at-risk mice. When we compared the gene expression changes of these fibroblast-like pericytes in control and at-risk aorta, we found an increased expression of pro-inflammatory genes, including *CXCL12*, *CXCL13*, *CCL1*, *IL1* and *IL6* (Fig. S15b)*.*

Immune cells (Figs S16–18) were more heterogeneous in at-risk than healthy mice, and we identified pro-inflammatory cells: Th17 cells (*CD4*, *CCR6* and *IL17*), cytotoxic CD8^+^ T cells (*CD8* and *IFNG*), macrophages expressing high levels of pro-inflammatory chemokines (*CXCL10*, *CXCL2* and *CCL3*) and regulatory B cells (*CD24* and *IL10*). More interestingly, two pro-inflammatory subpopulations, cytotoxic CD8^+^ T cells and pro-atherogenic B2 cells [69], tended to be enriched in the abdominal segment in all three at-risk groups.

Finally, we found that the population of SMC-like *LY6A^high^* mesothelial cells was markedly diminished in aortas from all at-risk mice (Fig. S19).

#### Intercellular network in at-risk abdominal aorta

We explored the changes in cell–cell interaction between healthy and all stress-treated mice using CellPhone DB. Results showed an extensive decrease in the interaction between SMCs *versus* ECs or stromal cells, and these three types of cells also showed diminished interaction within their own subtypes (Fig. S20). These changes may lead to further exploration for key signaling in vascular risk factor-induced lesions.

Furthermore, from the above results, we found that the abdominal aorta responded dramatically to metabolic pathology with significant changes in the subpopulations of ECs, macrophages and fibroblasts. Gene expression profiles of these cells all exhibited pro-inflammatory features with immune system activation (Fig. S21a and b). From the expression changes of certain genes, we conclude that in the abdominal aorta, extracellular matrix synthesis (by genes like *COL1A1* and *COL1A2*) was de-regulated, and matrix metalloproteinase expression (i.e. MMP3) was enhanced in fibroblasts, leading to the destruction of aortic architecture. Second, EC homeostasis was damaged, with increased apoptotic signaling and a pro-contractile phenotype (i.e. increased *SERPINE1* and *ACE* along with decreased *FLT1* and *KDR*).

We also found that the *IRF9*, *NR2C2* and *ATF1* regulons were predominantly activated in abdominal at-risk ECs, macrophages and fibroblasts (Fig. 5b). Finally, we constructed the gene network by calculating the correlations of the top differentially expressed genes in ECs, macrophages and fibroblasts after pathogenic change (Fig. S21c). We found that three types of these cells were tightly connected with the pluripotency subnetwork. For example, we identified a subnetwork involving endothelial *IL33* ↔ macrophage *CXCL13* ↔ fibroblast *CDKN1A*; each component within this subnetwork is associated with vasculopathy [70–72], and here we suggest that they interact during metabolic pathology in the aorta.

## DISCUSSION

In this study, we revealed the cell composition of the mouse aorta, resolved the cellular and functional heterogeneity of aortic cells among segments, uncovered the intercellular networks between these aortic cells and determined the effects of cardiovascular risk factors on aortic cell composition.

A recent scRNA-Seq study provided the first atlas of aortic cells [3]. In this report, mainly ECs and fibroblasts were obtained and profiled with a cell count of ∼1000. Here, we further characterized previously unidentified aortic cell types, such as SMCs and pericytes. In addition, we extended knowledge of the spatial heterogeneity of aortic cells across the four anatomical segments, and of the contribution of the spatiotemporal complexity of shear stress to the vascular heterogeneity.

It is well recognized that spatiotemporal differences in shear stress determine the regional heterogeneity of EC phenotypes [59]. Using scRNA-Seq, we characterized three EC subpopulations with specific gene expression profiles and anatomical distribution in the aorta. Functionally, one of the EC subpopulations, activated ECs, may participate in the response to variations in shear stress and the regulation of vascular tone. These activated ECs were most abundant in the thoracic aorta, which is consistent with the idea that the thoracic segment acts to adapt the axial velocity and vorticity propagated from the aortic arch with the help of EC-induced dilation [73].

Shear-induced mechanotransduction not only contributes to the distribution of ECs, we also demonstrated that all aortic cells are heterogeneously distributed. Typically, the curvature of the aortic arch results in a distinct cellular composition. This segment, which is innervated by numbers of neuron cells that are surrounded by Schwann cells, senses changes in heart rate and blood pressure. Contractile SMCs densely populated the aortic arch, which may generate greater contractile power. Such muscle tone is essential to fill the three arteries that branch off the aortic arch, and is also necessary to push the blood flow lower in the body. Further, abundant pericytes may stabilize aortic arch cells under high shear and axial stress [74–77]. One well-studied mechanism is the strong expression of the *ANGPT1* gene in pericytes (Log_2_FC = 2.5 in aortic arch pericytes) encoding angiopoietin 1. Angiopoietin 1 binds to its receptor Tie2 both in ECs and pericytes, and this leads to stabilization of ECs [78,79].

In addition to biomechanical factors, variations in the biochemical composition of the blood contribute to aortic cell heterogeneity. High blood sugar/glucose levels and high dietary salt or fat intake are linked to a greater risk of vascular disease [64–67]. They generate pathogenic biochemical microenvironments that affect the cellular populations and their functions. For example, Cochain *et al.* and Winkels *et al.* have reported interesting work on aortic macrophage and leukocyte heterogeneity from mice fed on chow and a western diet [7,8], with which our results are consistent. These studies and ours showed a diverse phenotype of aortic immune cells. All four studies reported the appearance of pro-inflammatory Th17 cells, cytotoxic CD8^+^ T cells, inflammatory macrophages and regulatory B cells in the aorta after challenge with an unhealthy diet, while these subpopulations were absent or at low proportions in healthy aorta. In contrast, Cochain *et al.* and Winkels *et al.* [7,8] sorted CD45^+^ immune cells for scRNA-Seq, so they were able to detect rare immune cells such as *TREM2*^high^ macrophages, *CD11c*^high^ foamy macrophage, and two T cell subpopulations with inverse *CD5* and *LYC6C* expression. On the other hand, our results are complementary. We investigated the immune cells among the pool of aortic cells, thus providing an overview of their compositional change; we also showed the spatial heterogeneity of immune cells, for example, B1 cells, naïve T/Tregs and M2-macrophages were all strongly localized to the aortic arch, which may help to maintain its homeostasis under high axial and shear stress. We further explored the common features among mice fed a high-salt diet, a high-fat diet or with high blood glucose, and found that inflammatory macrophages and T cells were most likely to be enriched in the abdominal aorta. It is particularly important to note that although single-cell sequencing data are valuable for the analysis of shifts in gene expression within diverse populations, it is not the best approach for estimating the proportions of cell populations. In this study, we used digested intact aorta to ensure that the sequenced single-cell suspension was close to the actual aortic cell composition, while also validating some of the conclusions about changes in cell composition using immunofluorescence. However, more precise methods, such as fluorescence-activated cell sorting, are required to confirm or deny the actual cell composition indicted in our study.

The responses of cells to pathogenic microenvironments were common to the three at-risk conditions to a certain extent, and distinct in different aortic segments. Of note, the abdominal aorta was the most changed segment under at-risk conditions that induce vasculopathy. *OCLN*^low^*SERPINE1*^high^ ECs, contractile SMCs, pro-inflammatory fibroblast-like pericytes, cytotoxic CD8^+^ T cells, macrophages and pro-atherogenic B2 cells all tended to accumulate in the abdominal segment, possibly to promote inflammation and reduce dilation. Also, ECs, fibroblasts and macrophages showed intensive communication during the pathological process in the abdominal aorta. These findings are in accordance with the high clinical incidence of aneurysms, dissection and atherosclerosis in the abdominal aorta.

Overall, our results provide a comprehensive transcriptome profile of single cells in the aorta (Fig. 5c), including cell-type-specific markers and intercellular networks, and this could lead to new means of diagnosis and intervention in metabolic pathologies of the aorta.

## MATERIALS AND METHODS

All data and methods used in the analysis, and information on materials used to conduct the research are available in the online-only supplement.

## Supplementary Material

nwaa038_supplemental_file

## References

[1] Yeager ME , Frid MG, Stenmark KR. Progenitor cells in pulmonary vascular remodeling. Pulm Circ 2011; 1: 3–16. 22034593 10.4103/2045-8932.78095 PMC3198626

[2] Benjamin M , Ralphs JR. Biology of fibrocartilage cells. Int Rev Cytol 2004; 233: 1–45. 15037361 10.1016/S0074-7696(04)33001-9

[3] Tabula Muris C , Overall C, Logistical C et al. Single-cell transcriptomics of 20 mouse organs creates a Tabula Muris. Nature 2018; 562: 367–72. 30283141 10.1038/s41586-018-0590-4 PMC6642641

[4] Haber AL , Biton M, Rogel N et al. A single-cell survey of the small intestinal epithelium. Nature 2017; 551: 333–9. 29144463 10.1038/nature24489 PMC6022292

[5] Kotchen TA , Cowley AW Jr, Frohlich ED. Salt in health and disease–a delicate balance. N Engl J Med 2013; 368: 2531–2. 10.1056/NEJMra1212606 23534562

[6] Chen JY , Tsai PJ, Tai HC et al. Increased aortic stiffness and attenuated lysyl oxidase activity in obesity. Arterioscler Thromb Vasc Biol 2013; 33: 839–46. 23413430 10.1161/ATVBAHA.112.300036

[7] Cochain C , Vafadarnejad E, Arampatzi P et al. Single-Cell RNA-Seq reveals the transcriptional landscape and heterogeneity of aortic macrophages in murine atherosclerosis. Circ Res 2018; 122: 1661–74. 29545365 10.1161/CIRCRESAHA.117.312509

[8] Winkels H , Ehinger E, Vassallo M et al. Atlas of the immune cell repertoire in mouse atherosclerosis defined by single-cell RNA-Sequencing and mass cytometry. Circ Res 2018; 122: 1675–88. 29545366 10.1161/CIRCRESAHA.117.312513 PMC5993603

[9] Breviario F , Caveda L, Corada M et al. Functional properties of human vascular endothelial cadherin (7B4/cadherin-5), an endothelium-specific cadherin. Arterioscler Thromb Vasc Biol 1995; 15: 1229–39. 7627717 10.1161/01.atv.15.8.1229

[10] Newman PJ , Berndt MC, Gorski J et al. PECAM-1 (CD31) cloning and relation to adhesion molecules of the immunoglobulin gene superfamily. Science 1990; 247: 1219–22. 1690453 10.1126/science.1690453

[11] Rensen SS , Doevendans PA, van Eys GJ. Regulation and characteristics of vascular smooth muscle cell phenotypic diversity. Netherlands Heart J 2007; 15: 100–8. 10.1007/BF03085963 PMC1847757 17612668

[12] Nishida W , Kitami Y, Hiwada K. cDNA cloning and mRNA expression of calponin and SM22 in rat aorta smooth muscle cells. Gene 1993; 130: 297–302. 8359698 10.1016/0378-1119(93)90435-6

[13] Cochain C , Vafadarnejad E, Arampatzi P et al. Single-Cell RNA-Seq reveals the transcriptional landscape and heterogeneity of aortic macrophages in murine atherosclerosis. Circ Res 2018; 122: 1661–74. 29545365 10.1161/CIRCRESAHA.117.312509

[14] Lau SK , Chu PG, Weiss LM. CD163: a specific marker of macrophages in paraffin-embedded tissue samples. Am J Clin Pathol 2004; 122: 794–801. 15491976 10.1309/QHD6-YFN8-1KQX-UUH6

[15] Sweet MJ , Hume DA. CSF-1 as a regulator of macrophage activation and immune responses. Arch Immunol Ther Exp 2003; 51: 169–77. 12894871

[16] Sidney LE , Branch MJ, Dunphy SE et al. Concise review: evidence for CD34 as a common marker for diverse progenitors. Stem Cells 2014; 32: 1380–9. 24497003 10.1002/stem.1661 PMC4260088

[17] Anjos-Afonso F , Bonnet D. Prospective identification and isolation of murine bone marrow derived multipotent mesenchymal progenitor cells. Best Pract Res Clin Haematol 2011; 24: 13–24. 21396589 10.1016/j.beha.2010.11.003

[18] Li IMH , Horwell AL, Chu G et al. Characterization of mesenchymal-fibroblast cells using the Col1a2 Promoter/Enhancer. Methods Mol Biol 2017; 1627: 139–61. 28836200 10.1007/978-1-4939-7113-8_10

[19] Morikawa S , Mabuchi Y, Kubota Y et al. Prospective identification, isolation, and systemic transplantation of multipotent mesenchymal stem cells in murine bone marrow. J Exp Med 2009; 206: 2483–96. 19841085 10.1084/jem.20091046 PMC2768869

[20] Daneman R , Zhou L, Kebede AA et al. Pericytes are required for blood-brain barrier integrity during embryogenesis. Nature 2010; 468: 562–6. 20944625 10.1038/nature09513 PMC3241506

[21] Kanamori-Katayama M , Kaiho A, Ishizu Y et al. LRRN4 and UPK3B are markers of primary mesothelial cells. PLoS One 2011; 6: e25391. 21984916 10.1371/journal.pone.0025391 PMC3184985

[22] Chang K , Pastan I. Molecular cloning of mesothelin, a differentiation antigen present on mesothelium, mesotheliomas, and ovarian cancers. Proc Natl Acad Sci USA 1996; 93: 136–40. 8552591 10.1073/pnas.93.1.136 PMC40193

[23] Deng Y , Kim B, He X et al. Direct visualization of membrane architecture of myelinating cells in transgenic mice expressing membrane-anchored EGFP. Genesis 2014; 52: 341–9. 24851283 10.1002/dvg.22751 PMC4047524

[24] Doerflinger NH , Macklin WB, Popko B. Inducible site-specific recombination in myelinating cells. Genesis 2003; 35: 63–72. 12481300 10.1002/gene.10154

[25] Butcher MJ , Galkina EV. Phenotypic and functional heterogeneity of macrophages and dendritic cell subsets in the healthy and atherosclerosis-prone aorta. Front Physiol 2012; 3: 44. 22457649 10.3389/fphys.2012.00044 PMC3307136

[26] Gao S , Yan L, Wang R et al. Tracing the temporal-spatial transcriptome landscapes of the human fetal digestive tract using single-cell RNA-sequencing. Nat Cell Biol 2018; 20: 721–34. 29802404 10.1038/s41556-018-0105-4

[27] Wehrwein EA , Orer HS, Barman SM. Overview of the anatomy, physiology, and pharmacology of the autonomic nervous system. Compr Physiol 2016; 6: 1239–78. 27347892 10.1002/cphy.c150037

[28] Orekhov AN , Bobryshev YV, Chistiakov DA. The complexity of cell composition of the intima of large arteries: focus on pericyte-like cells. Cardiovasc Res 2014; 103: 438–51. 25016615 10.1093/cvr/cvu168

[29] Strilic B , Kucera T, Eglinger J et al. The molecular basis of vascular lumen formation in the developing mouse aorta. Dev Cell 2009; 17: 505–15. 19853564 10.1016/j.devcel.2009.08.011

[30] Kim JY , Choi JS, Song SH et al. Stem cell factor is a potent endothelial permeability factor. Arterioscler Thromb Vasc Biol 2014; 34: 1459–67. 24790137 10.1161/ATVBAHA.114.303575

[31] Yoder MC . Endothelial stem and progenitor cells (stem cells): (2017 Grover Conference Series). Pulm Circ 2018; 8: 2045893217743950. 29099663 10.1177/2045893217743950 PMC5731724

[32] Lukowski SW , Patel J, Andersen SB et al. Single-Cell transcriptional profiling of aortic endothelium identifies a hierarchy from endovascular progenitors to differentiated cells. Cell Rep 2019; 27: 2748–58. 31141696 10.1016/j.celrep.2019.04.102

[33] Kalluri AS , Vellarikkal SK, Edelman ER et al. Single-cell analysis of the normal mouse aorta reveals functionally distinct endothelial cell populations. Circulation 2019; 140: 147–63. 31146585 10.1161/CIRCULATIONAHA.118.038362 PMC6693656

[34] Shanahan CM , Weissberg PL. Smooth muscle cell heterogeneity: patterns of gene expression in vascular smooth muscle cells *in vitro* and *in vivo*. Arterioscler Thromb Vasc Biol 1998; 18: 333–8. 9514400 10.1161/01.atv.18.3.333

[35] Paul RJ , Bauer M, Pease W. Vascular smooth muscle: aerobic glycolysis linked to sodium and potassium transport processes. Science 1979; 206: 1414–6. 505014 10.1126/science.505014

[36] Seiffert D , Iruela-Arispe ML, Sage EH et al. Distribution of vitronectin mRNA during murine development. Dev Dyn 1995; 203: 71–9. 7544171 10.1002/aja.1002030108

[37] Dahm LM , Bowers CW. Vitronectin regulates smooth muscle contractility via alphav and beta1 integrin. J Cell Sci 1998; 111: 1175–83. 9547294 10.1242/jcs.111.9.1175

[38] Dufourcq P , Louis H, Moreau C et al. Vitronectin expression and interaction with receptors in smooth muscle cells from human atheromatous plaque. Arterioscler Thromb Vasc Biol 1998; 18: 168–76. 9484980 10.1161/01.atv.18.2.168

[39] Cai Y , Nagel DJ, Zhou Q et al. Role of cAMP-phosphodiesterase 1C signaling in regulating growth factor receptor stability, vascular smooth muscle cell growth, migration, and neointimal hyperplasia. Circ Res 2015; 116: 1120–32. 25608528 10.1161/CIRCRESAHA.116.304408 PMC4702253

[40] Arnold C , Feldner A, Pfisterer L et al. RGS5 promotes arterial growth during arteriogenesis. EMBO Mol Med 2014; 6: 1075–89. 24972930 10.15252/emmm.201403864 PMC4154134

[41] Davis BN , Hilyard AC, Nguyen PH et al. Induction of microRNA-221 by platelet-derived growth factor signaling is critical for modulation of vascular smooth muscle phenotype. J Biol Chem 2009; 284: 3728–38. 19088079 10.1074/jbc.M808788200 PMC2635044

[42] Wang CH , Verma S, Hsieh IC et al. Stem cell factor attenuates vascular smooth muscle apoptosis and increases intimal hyperplasia after vascular injury. Arterioscler Thromb Vasc Biol 2007; 27: 540–7. 17204664 10.1161/01.ATV.0000257148.01384.7d

[43] Huang J , Elicker J, Bowens N et al. Myocardin regulates BMP10 expression and is required for heart development. J Clin Invest 2012; 122:3678–91. 22996691 10.1172/JCI63635 PMC3461917

[44] Keeley EC , Mehrad B, Strieter RM. The role of fibrocytes in fibrotic diseases of the lungs and heart. Fibrogenesis Tissue Repair 2011; 4: 2. 21219601 10.1186/1755-1536-4-2 PMC3027110

[45] Elahy M , Baindur-Hudson S, Dass CR. The emerging role of PEDF in stem cell biology. J Biomed Biotechnol 2012; 2012: 239091. 22675247 10.1155/2012/239091 PMC3362874

[46] Ma S , Wang S, Li M et al. The effects of pigment epithelium-derived factor on atherosclerosis: putative mechanisms of the process. Lipids Health Dis 2018; 17: 240. 30326915 10.1186/s12944-018-0889-z PMC6192115

[47] Rychli K , Kaun C, Hohensinner PJ et al. The anti-angiogenic factor PEDF is present in the human heart and is regulated by anoxia in cardiac myocytes and fibroblasts. J Cell Mol Med 2010; 14: 198–205. 19298519 10.1111/j.1582-4934.2009.00731.x PMC2883745

[48] Zheng C , Zheng L, Yoo JK et al. Landscape of infiltrating T cells in liver cancer revealed by single-cell sequencing. Cell 2017; 169: 1342–56. 28622514 10.1016/j.cell.2017.05.035

[49] Kyaw T , Tay C, Krishnamurthi S et al. B1a B lymphocytes are atheroprotective by secreting natural IgM that increases IgM deposits and reduces necrotic cores in atherosclerotic lesions. Circ Res 2011; 109: 830–40. 21868694 10.1161/CIRCRESAHA.111.248542

[50] Dettman RW , Denetclaw W Jr, Ordahl CP et al. Common epicardial origin of coronary vascular smooth muscle, perivascular fibroblasts, and intermyocardial fibroblasts in the avian heart. Dev Biol 1998; 193: 169–81. 9473322 10.1006/dbio.1997.8801

[51] Que J , Wilm B, Hasegawa H et al. Mesothelium contributes to vascular smooth muscle and mesenchyme during lung development. Proc Natl Acad Sci USA 2008; 105: 16626–30. 18922767 10.1073/pnas.0808649105 PMC2567908

[52] Zhou B , Ma Q, Rajagopal S et al. Epicardial progenitors contribute to the cardiomyocyte lineage in the developing heart. Nature 2008; 454: 109–13. 18568026 10.1038/nature07060 PMC2574791

[53] Efremova M , Vento-Tormo M, Teichmann SA et al. CellPhoneDB v2.0: Inferring cell-cell communication from combined expression of multi-subunit receptor-ligand complexes. bioRxiv 2019:680926. 10.1038/s41596-020-0292-x 32103204

[54] Ajami NE , Gupta S, Maurya MR et al. Systems biology analysis of longitudinal functional response of endothelial cells to shear stress. Proc Natl Acad Sci USA 2017; 114: 10990–5. 28973892 10.1073/pnas.1707517114 PMC5642700

[55] Buckley CD , Rainger GE, Nash GB et al. Endothelial cells, fibroblasts and vasculitis. Rheumatology 2005; 44: 860–3. 15644388 10.1093/rheumatology/keh542 PMC3119433

[56] Karra R , Walter AO, Wu SM. The relationship between cardiac endothelium and fibroblasts: it's complicated. J Clin Invest 2017; 127: 2892–4. 28650344 10.1172/JCI95492 PMC5531567

[57] Ramilowski JA , Goldberg T, Harshbarger J et al. A draft network of ligand-receptor-mediated multicellular signalling in human. Nat Commun 2015; 6: 7866. 26198319 10.1038/ncomms8866 PMC4525178

[58] Rahimi N . VEGFR-1 and VEGFR-2: two non-identical twins with a unique physiognomy. Front Biosci 2006; 11: 818–29. 16146773 10.2741/1839 PMC1360224

[59] Davies PF . Hemodynamic shear stress and the endothelium in cardiovascular pathophysiology. Nature Clin Pract Cardiovasc Med 2009; 6: 16–26. 19029993 10.1038/ncpcardio1397 PMC2851404

[60] Dai G , Vaughn S, Zhang Y et al. Biomechanical forces in atherosclerosis-resistant vascular regions regulate endothelial redox balance via phosphoinositol 3-kinase/Akt-dependent activation of Nrf2. Circ Res 2007; 101: 723–33. 17673673 10.1161/CIRCRESAHA.107.152942

[61] Dekker RJ , van Soest S, Fontijn RD et al. Prolonged fluid shear stress induces a distinct set of endothelial cell genes, most specifically lung Kruppel-like factor (KLF2). Blood 2002; 100: 1689–98. 12176889 10.1182/blood-2002-01-0046

[62] Ziegler T , Bouzourene K, Harrison VJ et al. Influence of oscillatory and unidirectional flow environments on the expression of endothelin and nitric oxide synthase in cultured endothelial cells. Arterioscler Thromb Vasc Biol 1998; 18: 686–92. 9598825 10.1161/01.atv.18.5.686

[63] Ley K . The role of selectins in inflammation and disease. Trends Mol Med 2003; 9: 263–8. 12829015 10.1016/s1471-4914(03)00071-6

[64] Bibbins-Domingo K , Chertow GM, Coxson PG et al. Projected effect of dietary salt reductions on future cardiovascular disease. N Engl J Med 2010; 362: 590–9. 20089957 10.1056/NEJMoa0907355 PMC3066566

[65] Watts GF , Lewis B, Brunt JN et al. Effects on coronary artery disease of lipid-lowering diet, or diet plus cholestyramine, in the St Thomas' Atherosclerosis Regression Study (STARS). Lancet 1992; 339: 563–9. 1347091 10.1016/0140-6736(92)90863-x

[66] Dimmeler S . Cardiovascular disease review series. EMBO Mol Med 2011; 3: 697. 22113984 10.1002/emmm.201100182 PMC3377116

[67] Johnson RK , Appel LJ, Brands M et al. Dietary sugars intake and cardiovascular health: a scientific statement from the American Heart Association. Circulation 2009; 120: 1011–20. 19704096 10.1161/CIRCULATIONAHA.109.192627

[68] Vaughan DE . PAI-1 and atherothrombosis. J Thromb Haemost 2005; 3: 1879–83. 16102055 10.1111/j.1538-7836.2005.01420.x

[69] Kyaw T , Tipping P, Toh BH et al. Current understanding of the role of B cell subsets and intimal and adventitial B cells in atherosclerosis. Curr Opin Lipidol 2011; 22: 373–9. 21881498 10.1097/MOL.0b013e32834adaf3

[70] Carlsen HS , Baekkevold ES, Morton HC et al. Monocyte-like and mature macrophages produce CXCL13 (B cell-attracting chemokine 1) in inflammatory lesions with lymphoid neogenesis. Blood 2004; 104: 3021–7. 15284119 10.1182/blood-2004-02-0701

[71] Miller AM . Role of IL-33 in inflammation and disease. J Inflamm 2011; 8: 22. 10.1186/1476-9255-8-22 PMC3175149 21871091

[72] Zhu F , Li Y, Zhang J et al. Senescent cardiac fibroblast is critical for cardiac fibrosis after myocardial infarction. PLoS One 2013; 8: e74535. 24040275 10.1371/journal.pone.0074535 PMC3770549

[73] Vincent PE , Plata AM, Hunt AA et al. Blood flow in the rabbit aortic arch and descending thoracic aorta. J R Soc Interface 2011; 8: 1708–19. 21593030 10.1098/rsif.2011.0116 PMC3203481

[74] Armulik A , Abramsson A, Betsholtz C. Endothelial/pericyte interactions. Circ Res 2005; 97: 512–23. 16166562 10.1161/01.RES.0000182903.16652.d7

[75] Bergers G , Song S. The role of pericytes in blood-vessel formation and maintenance. Neuro Oncol 2005; 7: 452–64. 16212810 10.1215/S1152851705000232 PMC1871727

[76] Volz KS , Jacobs AH, Chen HI et al. Pericytes are progenitors for coronary artery smooth muscle. eLife 2015; 4: e10036. 26479710 10.7554/eLife.10036 PMC4728130

[77] von Tell D , Armulik A, Betsholtz C. Pericytes and vascular stability. Exp Cell Res 2006; 312: 623–9. 16303125 10.1016/j.yexcr.2005.10.019

[78] Suri C , Jones PF, Patan S et al. Requisite role of angiopoietin-1, a ligand for the TIE2 receptor, during embryonic angiogenesis. Cell 1996; 87: 1171–80. 8980224 10.1016/s0092-8674(00)81813-9

[79] Thurston G , Suri C, Smith K et al. Leakage-resistant blood vessels in mice transgenically overexpressing angiopoietin-1. Science 1999; 286: 2511–4. 10617467 10.1126/science.286.5449.2511

