# Supplementary material for: Aortic heterogeneity across segments and under high fat/salt/glucose conditions at the single-cell level
Source: Natl Sci Rev. 2020 Mar 9;7(5):881–96. doi: 10.1093/nsr/nwaa038 (PMC8289085; doi:10.1093/nsr/nwaa038)
Supplement: nwaa038_supplemental_file [file nwaa038_supplemental_file.pdf]

Supplemental information

Aortic Heterogeneity Across Segments and Under High Fat/Salt/Glucose Conditions at the Single-Cell Level

Dongxu He[1,#], Aiqin Mao[1,#], Chang–Bo Zheng[2,#], Hao Kan[1,#], Ka Zhang[1,#], Zhiming Zhang[3], Lei Feng[1], Xin Ma[1,*]

Affiliations:

[1]Wuxi School of Medicine and School of Food Science and Technology, Jiangnan University

[2]School of Pharmaceutical Sciences and Yunnan Key Laboratory of Pharmacology for Natural Products, Kunming Medical University, Kunming, China

[3]School of Biotechnology, Jiangnan University

[#]These authors contributed equally to this study

*To whom correspondence should be addressed: Xin Ma, Wuxi School of Medicine, Jiangnan University, Wuxi, China. E–mail: maxin@jiangnan.edu.cn.

PART I. Supplemental materials and methods

EXPERIMENTAL MODEL

Mice All animal experiments were conducted using protocols approved by the Animal Care Committee of Jiangnan University. C57Bl/6J mice were housed under specific-pathogen-free conditions. To avoid sex-dependent factors that affect the cellular composition of the cardiovascular system [80], 5-week-old male mice were used and randomly assigned to

treatment groups. For the high-salt diet model, a diet containing 8% NaCl was provided, while the normal salt diet (0.3% NaCl) served as control. Tail-cuff plethysmography (NIPB-2 blood pressure monitor, Columbus Instruments, Columbus, OH) was used to measure systolic and diastolic blood pressure [81, 82]. For the high-fat model, mice were fed a diet with 60% fat, and the normal diet (11% fat) served as control. Mice with high blood glucose were created by intraperitoneal injection of 70 mg/kg streptozotocin, and those with plasma glucose levels >16.8 mmol/L 72 h after injection were selected for subsequent experiments.

Aorta collection and lysis Mice were euthanized by CO_2 inhalation. Aortas were collected after intracardiac perfusion twice with phosphate-buffered saline (PBS) and quickly transferred to cold PBS. After removing the perivascular adipose tissue, each aorta was cut into ~1-mm pieces and incubated with heparin-PBS containing 0.2% type I collagenase (Worthington Biochemical Corp., Lakewood, NJ) and 200 U/ml DNase (Sigma, USA) at 37℃ for 20 min. Then the cell suspension was passed through a 40-μm strainer (STEMCELL Technologies China Co., Ltd, Shanghai) and washed once with PBS.

METHOD DETAILS

Single-cell RNA sequencing The cell viability in aortic suspensions was determined by trypan blue staining. 300-1000 cells/ml in PBS with 0.04% BSA was loaded onto a GemCode Single-Cell Instrument (10x Genomics, Pleasanton, CA) to generate a single-cell Gel bead in EMulsion (GEM, Single Cell 3' Library and Gel Bead Kit V2 (10x Genomics)) [83]. Single-cell RNA was barcoded through reverse transcription in individual GEMs with an S1000™ thermal cycler (Bio-Rad, USA) running the following program: 53℃for 45 min, 85℃for 5 min; held at

4℃ After reverse transcription, GEMs were broken and the single-strand cDNA was cleaned up with DynaBeads MyOne Silane Beads (Thermo Fisher Scientific, USA). cDNA was amplified on an S1000™ thermal cycler (Thermo Fisher Scientific) running the following program: 98℃ for 3 min, 98℃ for 15 s, 67℃ for 20 s, 72℃ for 60 s, 12 cycles; 72℃ for 1 min, and held at 4℃ After cDNA amplification, cDNA was cleaned with SPRIselect Reagent (Beckman Coulter) and the cDNA sample was loaded onto an Agilent Bioanalyzer High Sensitivity chip for qualitative analysis. Indexed sequencing libraries were constructed using the reagents in the Chromium™ Single Cell 3' Reagent Kits (10x Genomics) in the following steps: (1) fragmentation, end repair, and A-tailing; (2) end repair and A-tailed double-sided size selection with SPRIselect reagent; (3) adaptor ligation with an Adaptor Ligation Mix and then post-ligation cleanup with SPRIselect reagent; and (4) sample index PCR and cleanup. The Agilent Bioanalyzer High Sensitivity chip was used for qualitative analysis of the library. Sequencing was performed on an Illumina HiSeq X Ten platform. The accession number for the RNA-seq data reported in this paper is NCBI SRA: PRJNA489757.

Sequencing data pre-processing CellRanger software (10x Genomics) was then used to analyze the sequencing data and produce gene expression information for each cell [84]. The software converted Illumina basecall files to fastq format. The clear raw sequence data (fastq) was produced using the FASTX-Toolkit. Then the reads were aligned to the mouse mm10 transcriptome. The total UMI counts for each cell-associated barcode were normalized to the grand median UMI count per cell by a scaling factor (median reads per barcode / reads per barcode), and then principal component analysis (PCA) was used to reduce the dimensionality of the raw data. The output dataset was loaded into Cell Ranger

(10x Genomics) to generate graph-based and K-mean clusters in the PCA space. In this study, a graph-based algorithm was used in dimensionality-reduction to generate two-dimensional t-distributed stochastic neighbor embedding (t-SNE) plots. Single-cell expression data, including $\log_2$ fold-change (FC) values (negative binomial test) and mean UMIs (normalized by the size factor: total UMI count per cell / median UMI count per cell) were generated with CellRanger. For quality control, cells with >10% reads mapping to mitochondria, or with <700 or >15,000 UMI counts indicated low quality, and were removed from the analysis.

Immunofluorescence Slides of aortic tissue were fixed in 4% paraformaldehyde at room temperature for 30 min and permeabilized with 0.1% PBS-Triton. The samples were incubated with fetal bovine serum (VisTech) and 0.5% PBS-Triton at 37°Cfor 1 h and then with the primary antibodies anti-CD31 (ab9498), anti-myelin proteolipid protein 1 (PLP1, ab28486), anti-early growth response protein 1 (Egr1, ab194357), anti-alpha-smooth muscle actin (ab7817), anti-endothelial nitric oxide synthase (eNOS, ab76198), anti-CD3d (ab109531), anti-CD68 (ab125212), anti-PDGFRB (ab69506), anti-PDGFRA (ab203491), anti-vimentin (ab137321), and anti-BANK1 (ab229189) (all from Abcam, USA). Then the slides were incubated with the secondary antibodies AF568 donkey anti-rabbit and AF647 donkey anti-mouse (Invitrogen, USA). Immunofluorescence was assessed by confocal laser-scanning microscopy (Zeiss LSM 880, Zeiss Microscopy, Jena, Germany) [85]. The fluorescence values were analyzed using ImageJ software (developed at the National Institutes of Health). At least 100 cells were analyzed for each segment.

Wire myography Vascular reactivity of freshly isolated aortic arteries was studied using a myograph (Danish Myo Technology, Aarhus, Denmark) as described [86]. After mice were sacrificed, the aorta was rapidly removed and placed in oxygenated ice-cold Krebs solution that contained (mmol/L): 119 NaCl, 4.7 KCl, 2.5 $CaCl_2$, 1 $MgCl_2$, 25 $NaHCO_3$, 1.2 KH_2PO_4, and 11 D-glucose. Connective tissue and blood were removed and four segments (2 mm in length) were mounted in organ baths individually gassed with 95% O_2 and 5% CO_2 and maintained at 37°C. Changes in isometric tone of the rings were recorded. The rings were stretched to an optimal baseline tension of 3 mN. After 60 min of equilibration at baseline tension, 60 mM KCl (the NaCl in Krebs solution was substituted with an equimolar amount of KCl) was applied to confirm the contractility of the ring, washed in Krebs solution, and finally allowed to equilibrate for 30 min. Phenylephrine (Phe) (1 nmol/L to 1 µmol/L) was added cumulatively, or Phe (1 µmol/L) was used to produce a steady contraction and then acetylcholine (ACh) (1 nmol/L-10 µmol/L) was added cumulatively. The dose-dependent contractile response to Phe is expressed as a percentage of 60 mmol/L KCl-induced contraction, while relaxation responses to ACh are expressed as a percentage of the constriction induced by Phe (1 µmol/L).

Evans blue permeability assay Mice were anesthetized with 4% chloral hydrate and fixed on a plate. Evans blue dye (5 mg/kg 1% Sigma) was injected into the inferior vena cava to stain the aorta through the systemic circulation. After 30 min of incubation, the intact aorta was removed and photographed under a light microscope. To quantify the aortic permeability, free Evans blue was removed by PBS perfusion. Then the aorta was weighed and cut into pieces. The Evans blue in tissue was extracted with formamide overnight at 55°C and the

concentration of Evans blue was measured on a spectrophotometer at 620 nm. The Evans blue content per gram of tissue was calculated.

STATISTICAL ANALYSIS

Cell clustering Principal component analysis was used to decompose the expression matrix of each cell into linear components by CellRanger software implemented with the IRLBA R package. Then the cells were clustered with a graph-based clustering algorithm and visualized as t-SNE plots using CellRanger software. The cell types were determined using significantly up-regulated marker genes ($\log_2$ fold-change >1.5, $p < 0.05$ *versus* the other clusters by the negative binomial test) in a certain cluster in the t-SNE plot, which was visualized with either Loupe Cell Browser (10x Genomics) or in the cell expression matrix generated by CellRanger.

Identification of heterogeneity of different aortic cell types To characterize the heterogeneity within a given aortic cell type, we used following steps: (a) combining the expression matrixes of healthy aortic cells from aortic segments (Figs S2 and 3); alternatively, the cells of aortic segments from either high-salt, high-glucose, or high-fat mice were combined (Figs S8–14); (b) clustering the combined cells with a graph-based algorithm; (c) defining the type of each cluster based on known markers; (d) selecting the indicated cell types and re-clustering them with the graph-based algorithm with SEURAT [87], and visualized with t-SNE; and (e) identifying the 50-200 most variable genes to draw a hierarchically-clustered heatmap.

GO analysis To identify the potential functional pathway of a set of genes, GO enrichment analyses were performed using the DAVID 6.8 functional annotation tool [88]. Briefly, cell types and subpopulations were determined using the graph-based t-SNE plots. A gene expressed with a $\log_2$ FC value >1.5 (negative binomial test) and a p value <0.05 between the indicated cell types/subpopulations and all remaining groups was determined to be up-regulated between the cell types/subpopulations. Then the list of significantly changed genes served as the input of GO in DAVID. A significantly enriched functional pathway was determined by the enrichment score and p value generated by DAVID.

Gene regulatory network analysis Regulon matrices were generated by SCENIC and used to map gene-regulatory networks [89]. The scRNA-Seq data from the abdominal aorta in healthy, high-salt intake, high-fat intake, and high plasma glucose mice were combined, and then ECs, myeloid cells, and fibroblasts were extracted from the combined dataset with known marker genes – *PECAM1* for ECs, *CD163* for myeloid cells, and *PDGFRA* for fibroblasts – using CellRanger software. Gene-expression matrices in which rows corresponded to log-transformed gene values and columns corresponded to cells were produced for each cell type. Then the matrices were loaded into GENIE3 to infer transcription factor co-expression modules [37]. Co-expression modules with <30 genes were filtered out. RcisTarget was then used for *cis*-regulatory motif analyses to build regulons. We found 329 regulons in ECs, 315 in fibroblasts, and 317 in myeloid cells. The activity of the regulons in cells was then calculated as the area under the recovery curve by AUCell across the genes ranked by their expression values, and active regulons were determined by the AUCell default threshold. The binary regulon activity matrix was mapped to t-SNE plots with the Rtsne package.

Regulons IRF9, NR2C2, and ATF1 were chosen as they predominated in at-risk ECs, fibroblasts, and myeloid cells but were inactive in healthy cells.

To determine the potential for fibroblast-EC communication in the aorta, we used mouse orthologs of human ligand-receptor pairs designed by Ramilowski *et al*. from the BioMart database [57]. We defined one fibroblast or endothelial subpopulation as expressing a certain ligand or receptor if the gene was significantly higher in the subpopulation than in the other subpopulations with a $\log_2$ FC value >1.5 (negative binomial test) and $p < 0.05$. When fibroblasts and ECs were in communication, the ligand was expressed in the fibroblast subpopulation and the receptor was expressed in the EC subpopulation [80]. Then a ligand-receptor matrix of the cell identities, $\log_2$ FCs, and p values of genes was created, and the list of receptors in the matrix was used for GO analysis. A heatmap (representative in Fig. 3b) was then drawn with the online tool Heatmapper [90] using the genes most enriched in each GO analysis.

To identify EC-SMC interactions, we searched PubMed for possible EC-SMC signaling pathways. Then each molecule from either the EC or SMC pathway was attributed to a subpopulation if it was up-regulated in this subpopulation (FC value >1.5, $p < 0.05$) and used to draw Figure 3c.

Cell-cell interaction was analyzed with CellPhoneDB [53] framework. Since CellPhoneDB uses human genome annotations for receptors, ligands and their interactions, mouse genes were mapped to human orthologs (Ensembl) using the BioMart database. CellPhoneDB analysis was performed using the default parameters of the system. Total cell-cell interaction

strength between cell types was calculated by counting the number of significant cell-cell molecular interactions ($p < 0.05$). Gain and loss of cell-cell interactions was calculated by subtracting the pairwise interaction strength of healthy aorta from the pairwise interaction strength of at-risk aortas.

And then the gene expression correlations between were calculated with the Pairwise Spearman's correlations in SPSS, and then the correlation coefficients were used to draw a heatmap with Heatmapper [90].

PART II. Supplemental Figures

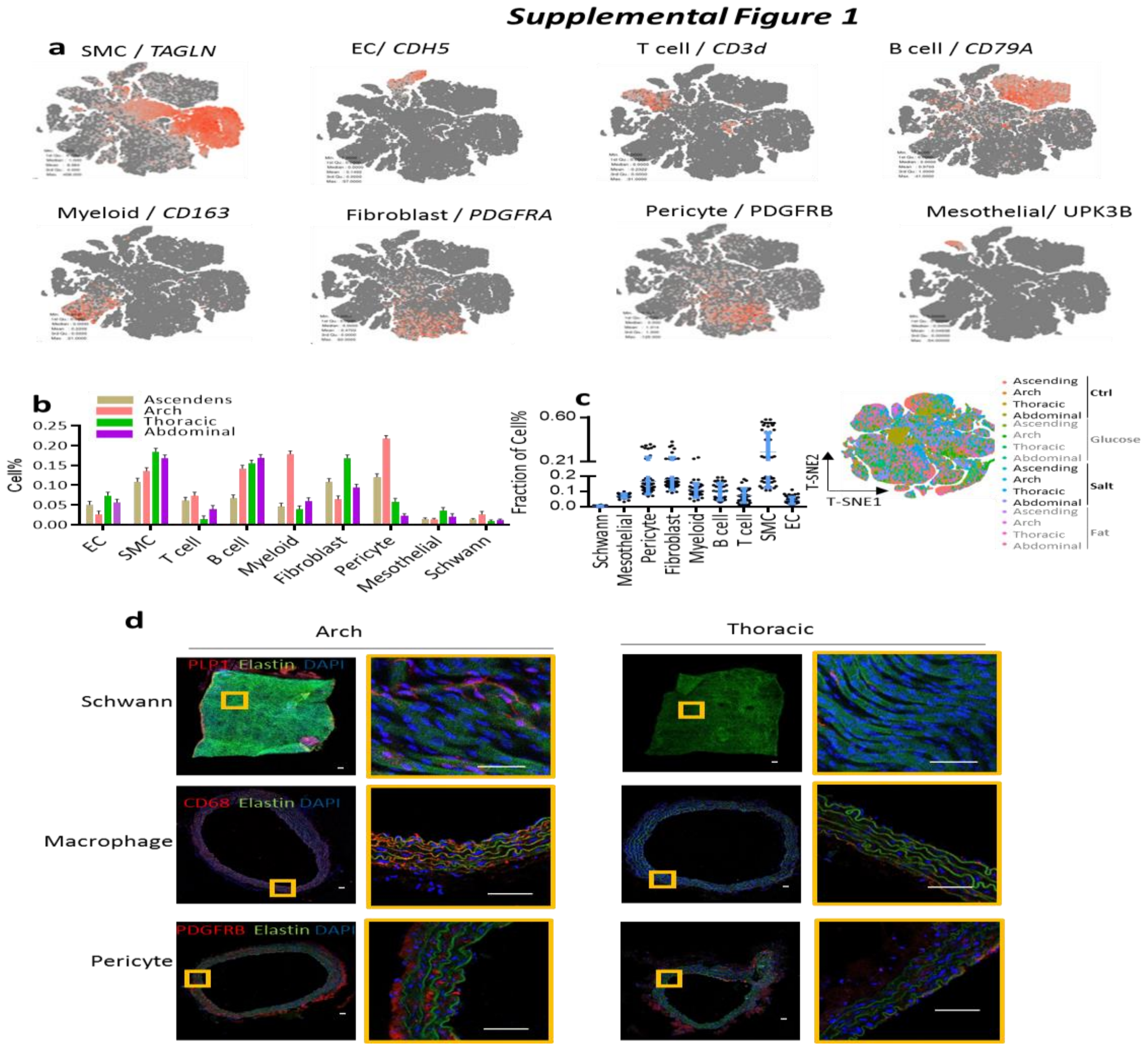

Supplemental Figure 1. Identifying aortic cell types in scRNA-seq data. **a.** t-SNEs showing the distribution of well-defined marker genes in single aortic cells. **b,** Cellular composition of each aortic segment, erythrocytes are not included. Data are shown as the mean ± s.e.m. **c.** Contribution of batches to each cluster. Dot-plot and t-SNE showing the batch composition of each detected cluster crossing four aortic segments from controls or at-risk aortas. All replicates contributed to all clusters, and no major batch effect was found. A total of 32 data from all aortic segments were used. Data are shown as the mean ± s.e.m. **d,** Immunofluorescence images comparing the distribution of Schwann cells, macrophages, and pericytes in aortic arch and thoracic segments. The thoracic segment was chosen because it had a lower percentage of these cell than the other segments. Scale bars, 50 µm.

Supplemental Figure 2

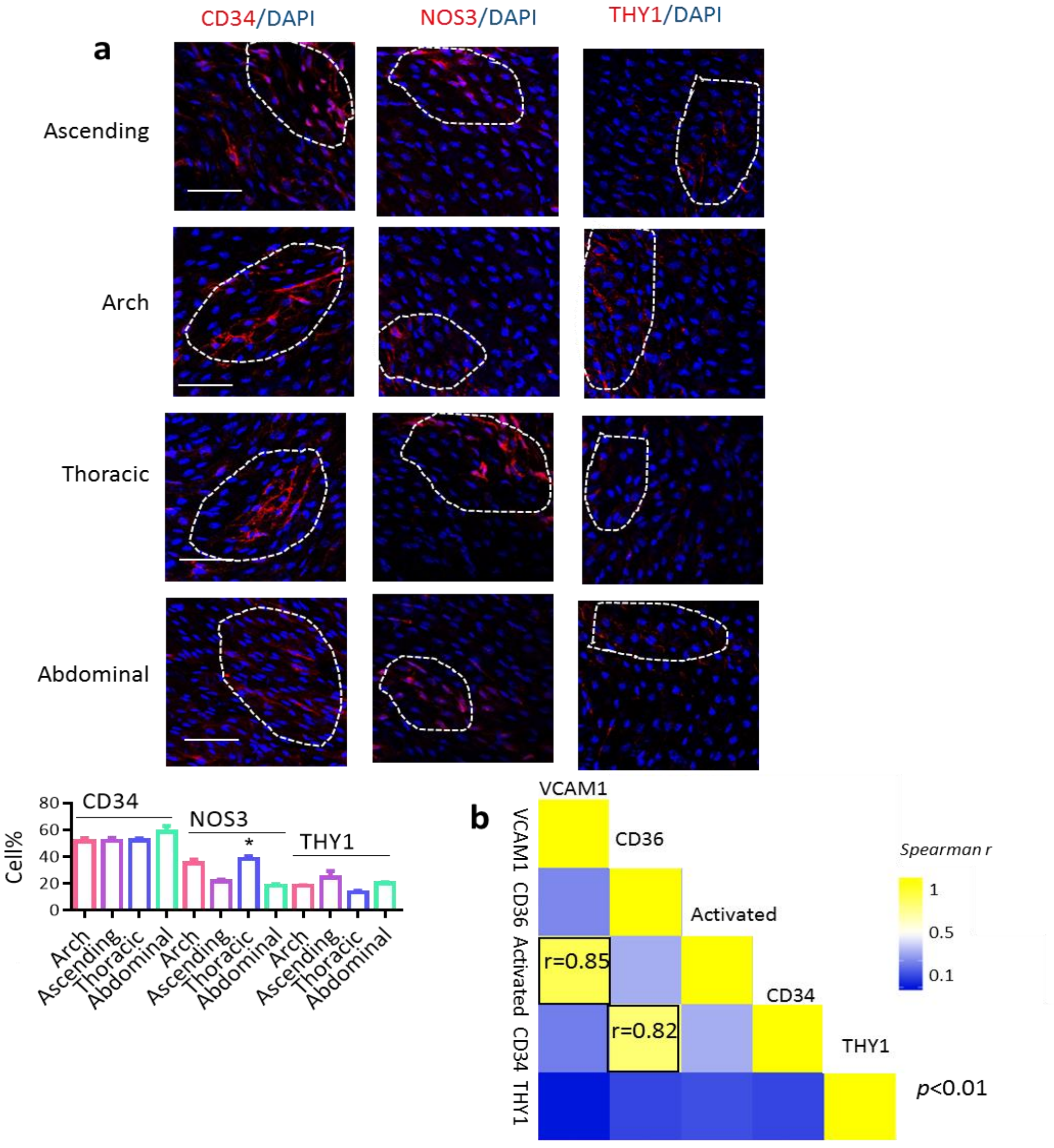

Supplemental Figure 2. Supplemental analysis for EC subpopulations. a, Immunofluorescence showing spatial heterogeneity of EC subpopulations. Data shows en-face image representing EC layer. Three subpopulations were labeled with respective markers: CD34 for contractile *CD34*^{high}; THY1 for synthetic *THY1*^{high}; NOS3 for activated EC. Quantitative analysis of immunofluorescence of four segments are shown in the bar graph. Scale bar, 50 µm, *p<0.05 *vs* ascending and abdominal. **b,** Comparison with previous studies. Gene expression of EC subtypes signatures from Kalluri *et al*'s study were calculated for Pairwise Spearman correlation with signatures of EC subtypes identified in this study. VCAM1 and CD36 represent EC subtypes identified in studies of Kalluri *et al*. Activated, *CD34*^{high} (CD34) and *THY*^{high} (THY1) are EC subtypes identified in this study.

Supplemental Figure 3

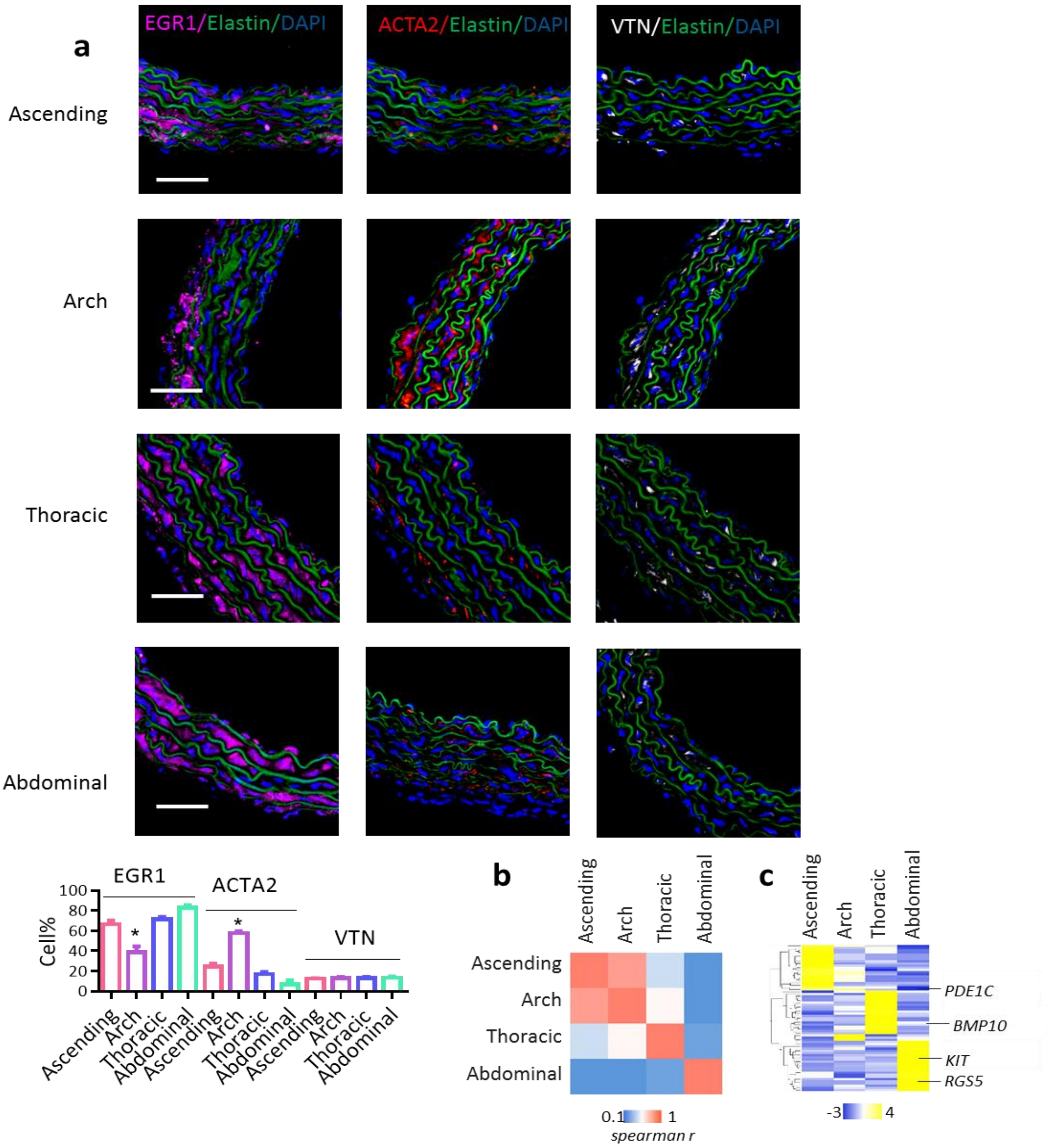

Supplemental Figure 3. Supplemental analysis for SMC subpopulations. a, Immunofluorescence showing spatial heterogeneity of SMC subpopulations. Three subpopulations were labeled with respective markers: ACTA2 for contractile SMC; EGR1 for synthetic SMC; VTN for VTN^{high} SMC. Quantitative analysis of immunofluorescence of four segments are shown in the bar graph. Scale bar, 50 µm, *p<0.05 *vs* other segments **b,** Gene expression profiles of synthetic SMCs from the four aortic segments were calculated for Pairwise Spearman correlation. **c,** Heatmap illustrating the top 20 genes most markedly changed in synthetic SMCs from the four aortic segments.

Supplemental Figure 4

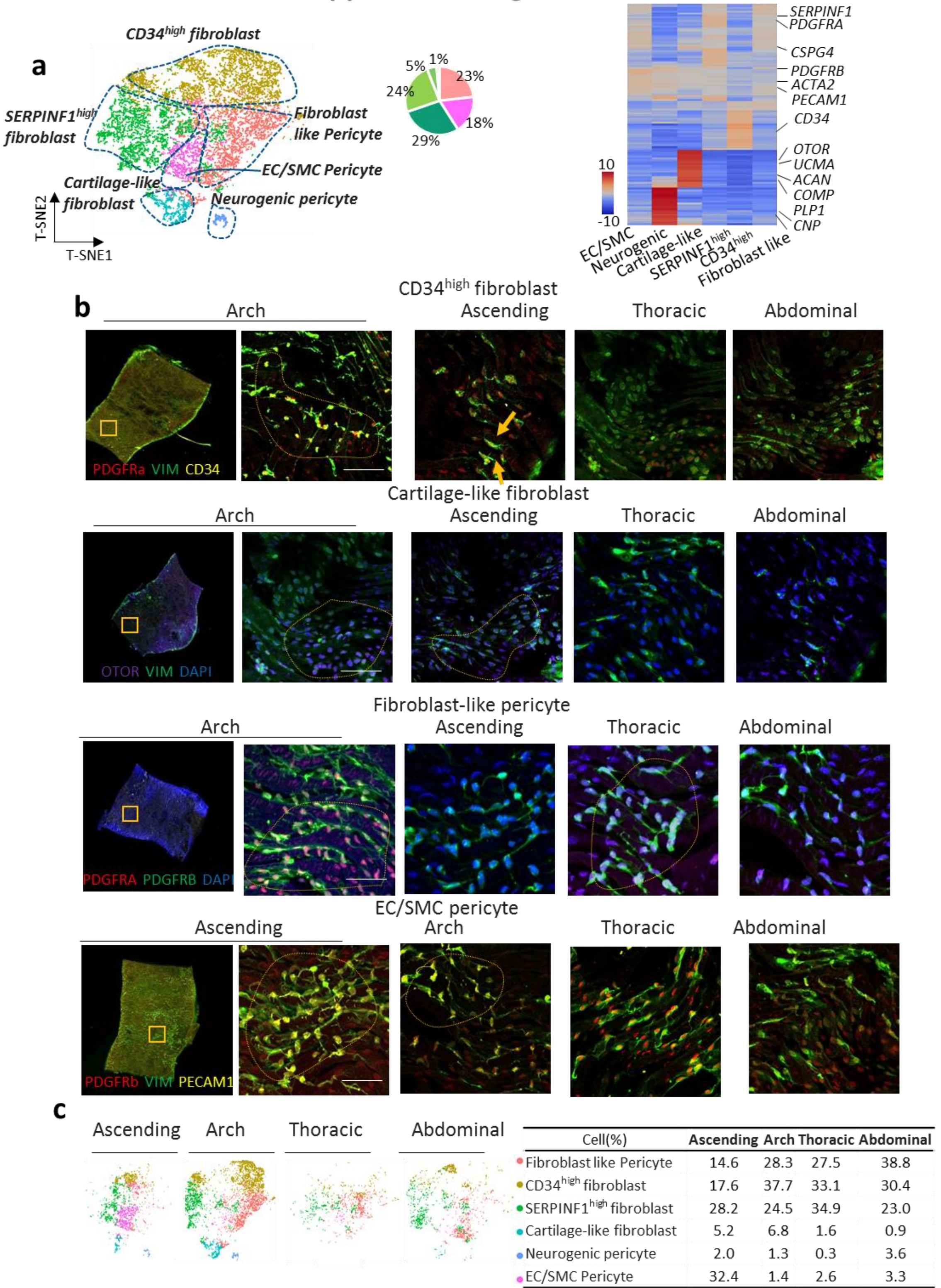

Cell(%)	Ascending	Arch	Thoracic	Abdominal
Fibroblast like Pericyte	14.6	28.3	27.5	38.8
CD34^{high} fibroblast	17.6	37.7	33.1	30.4
SERPINF1^{high} fibroblast	28.2	24.5	34.9	23.0
Cartilage-like fibroblast	5.2	6.8	1.6	0.9
Neurogenic pericyte	2.0	1.3	0.3	3.6
EC/SMC Pericyte	32.4	1.4	2.6	3.3

Supplemental Fig. 4. Subpopulations of stromal cells. a, t-SNEs of 12848 stromal cells (n = 8 mice each). Pie graph represents proportions of subpopulations. Heatmap shows 20 gene signatures for subpopulations. **b,** Immunostaining validation of stromal cell subpopulations across aortic segments. **c,** Regional distribution of subpopulations. The percentages of each subpopulation are summarized in the table. Scale bar, 50 µm.

Supplemental Figure 5

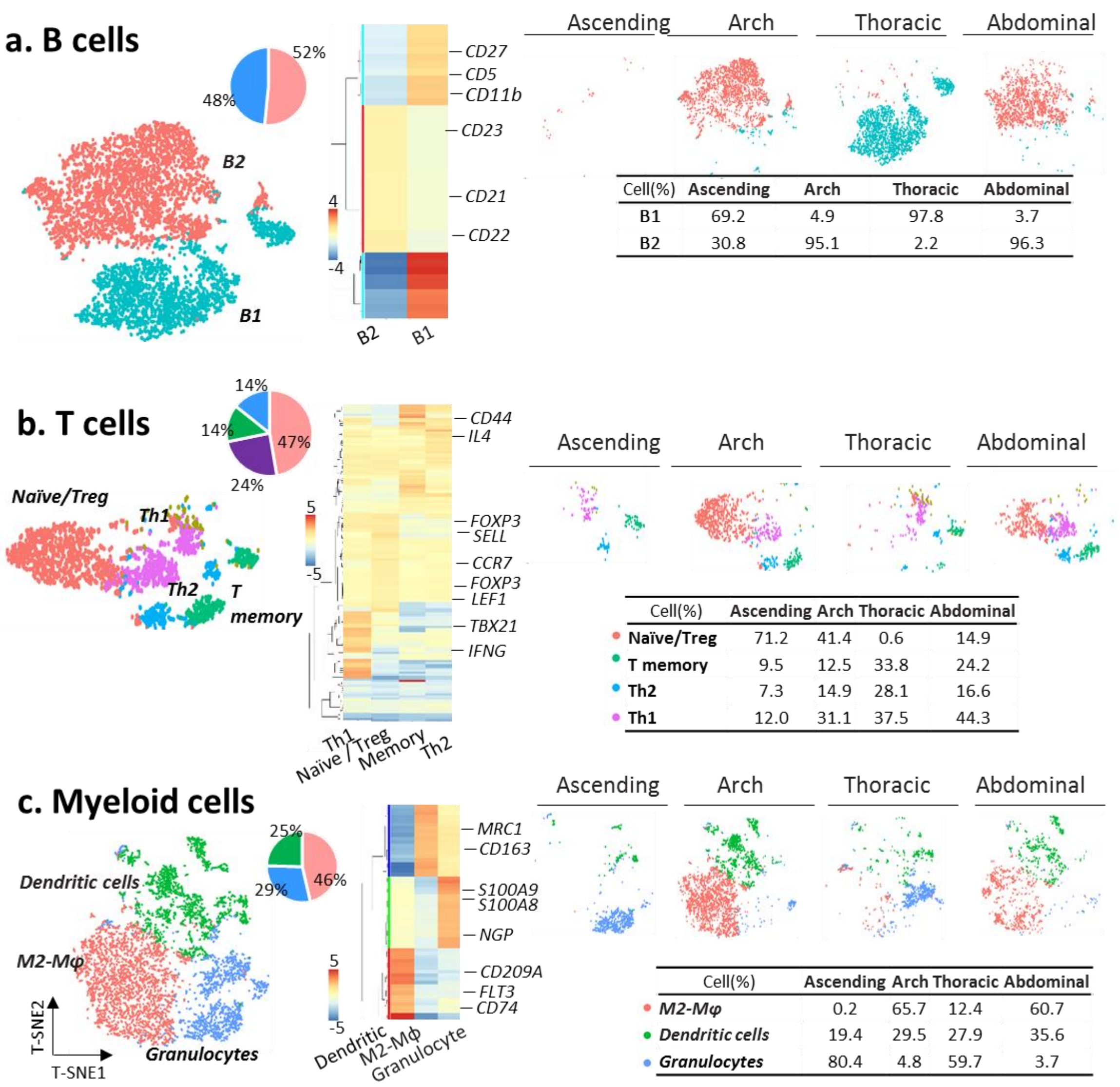

Supplemental Figure 5. Subpopulations of immune cells. Left, t-SNEs of 7031 B cells **(a)**, 2204 T cells **(b)**, and 6336 myeloid cells **(c)** (n = 8 mice each). Pie graphs represent proportions of subpopulations. Middle, 20 gene signatures for subpopulations. Right, regional distributions of subpopulations and summaries in tables.

Supplemental Figure 6

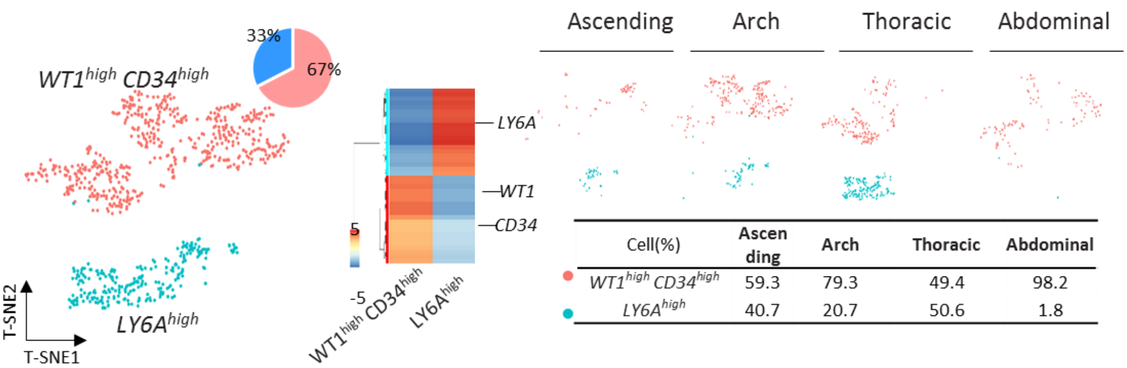

Cell(%)	Ascending	Arch	Thoracic	Abdominal
WT1^{high} *CD34*^{high}	59.3	79.3	49.4	98.2
LY6A^{high}	40.7	20.7	50.6	1.8

Supplemental Figure 6. Subpopulations of mesothelial cells. Left, t-SNEs of 1594 mesothelial cells. Pie graph represents proportions of subpopulations. Middle, 20 gene signatures for subpopulations. Right, regional distribution of subpopulations and summary in the table.

Supplemental Figure 7

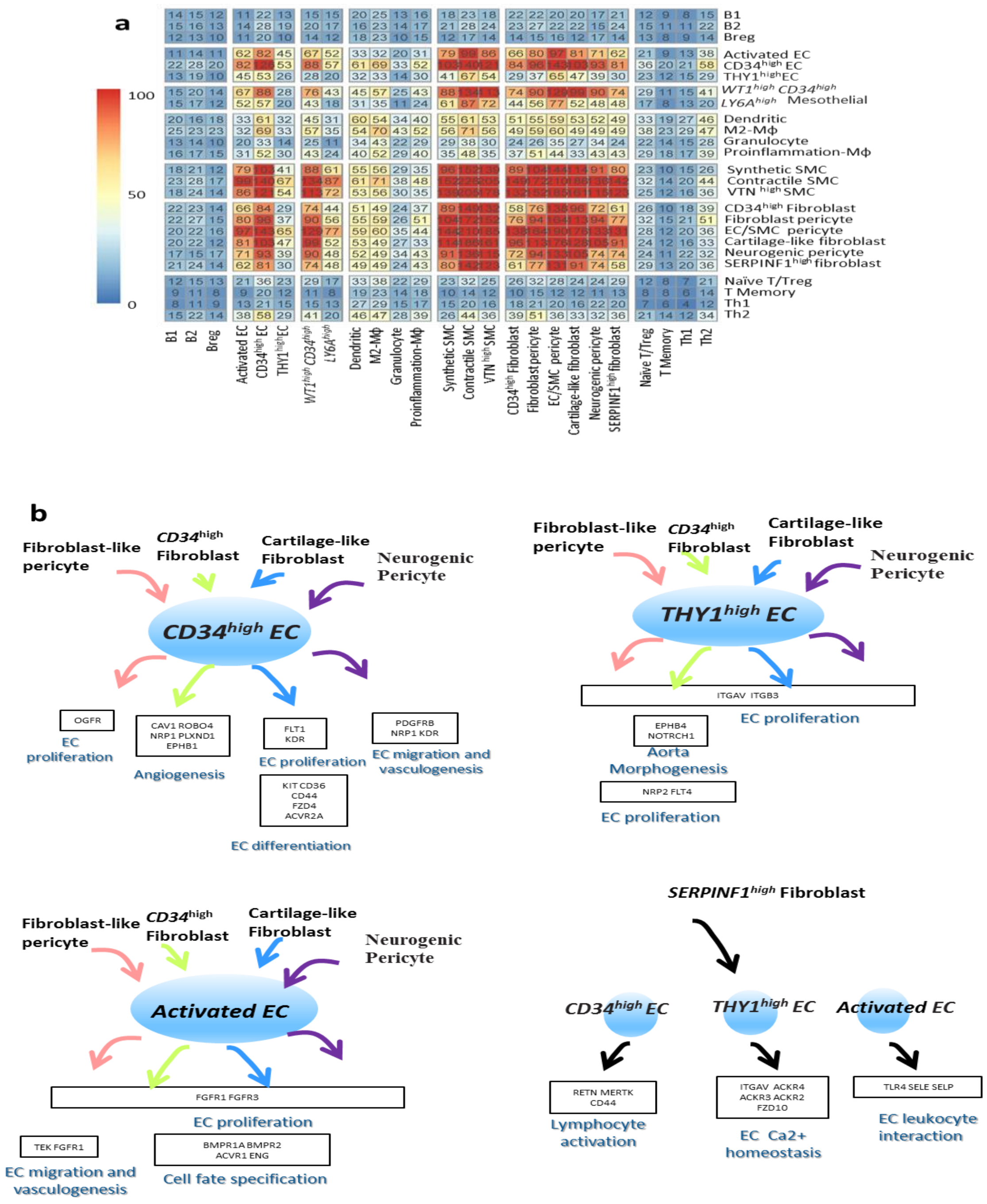

Supplemental Figure 7. Interaction between aortic cell subpopulations. a, Putative ligand and receptor-based cell-cell interaction between subpopulation of aortic cells. High value means strong cell-cell interaction. **b,** Related to Fig. 3b. Cartoon shows ligands from each subtype of stromal cell evoke highly-expressed receptors in different EC subpopulations. Certain stromal cells and their reciprocal receptors are indicated with arrows in the same colors.

Supplemental Figure 8

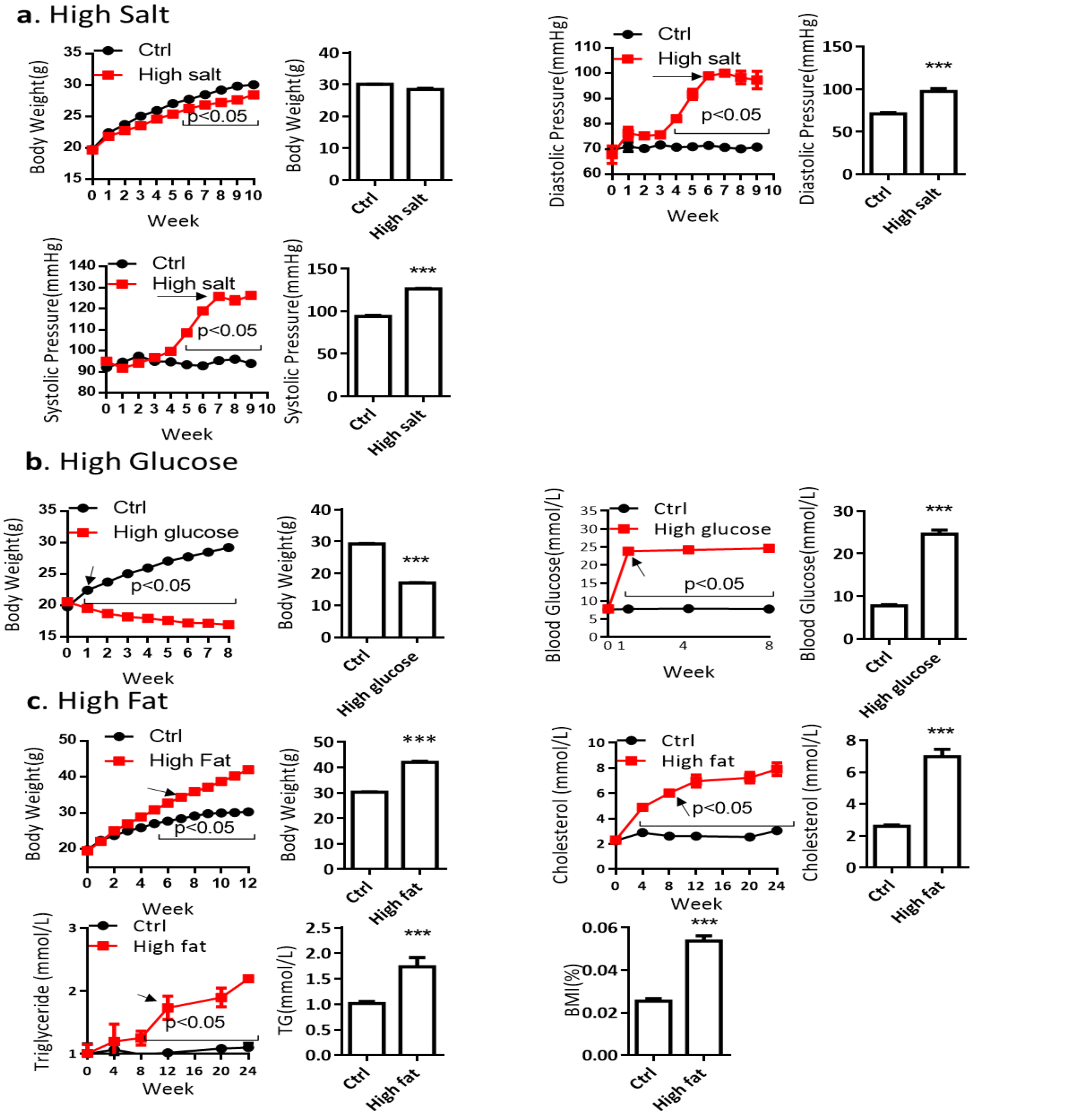

Supplemental Figure 8. Assessment of at-risk mice. a, Body weight and blood pressure during 10 weeks in mice on a high-salt diet. **b,** Body weight and blood glucose level during 8 weeks in mice with high blood glucose after intraperitoneal streptozotocin injection. **c,** Body weight, plasma cholesterol, plasma triglyceride, and body mass index during 24 weeks in mice on a high-fat diet. n = 10 for each analysis; Individual mice were chosen for aorta scRNA-Seq when the pathophysiological values described above reached relatively stable (but significant) levels as indicated by the line graphs in a-c and arrows. Bar graph, statistical analysis of physiological values on the chosen day.

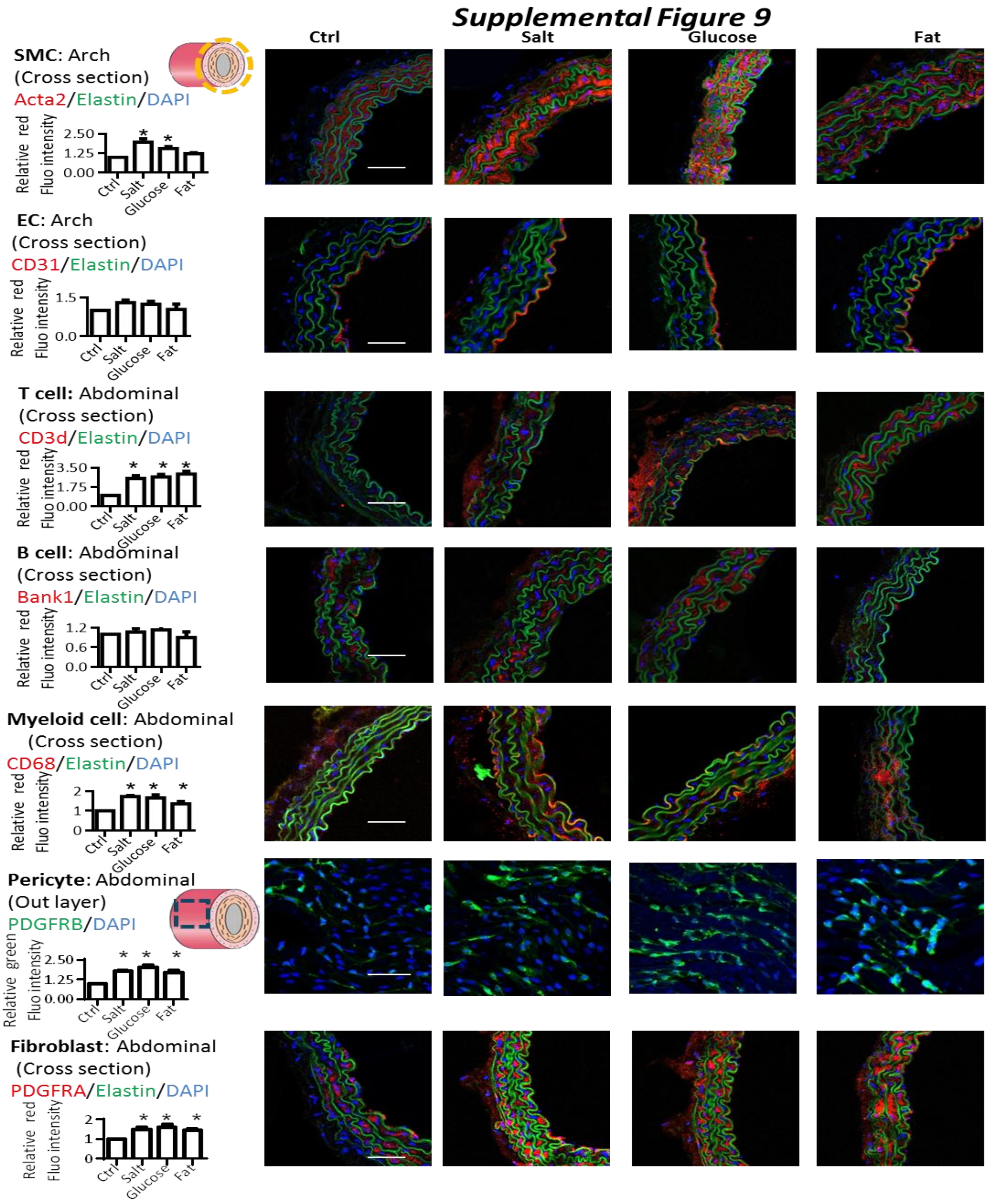

Supplemental Figure 9. Immunofluorescence validation of spatial heterogeneity of aortic cells. For each cell type, one aortic segment was chosen to be validated when this segment showed the most dramatic and typical cell change in composition according to Table S4. The statistical analysis of relative immunofluorescence values are shown in the bar graph, where the value for control aorta was normalized to 1. *p <0.05 *vs* control by one-way ANOVA. Scale bar, 50 µm.

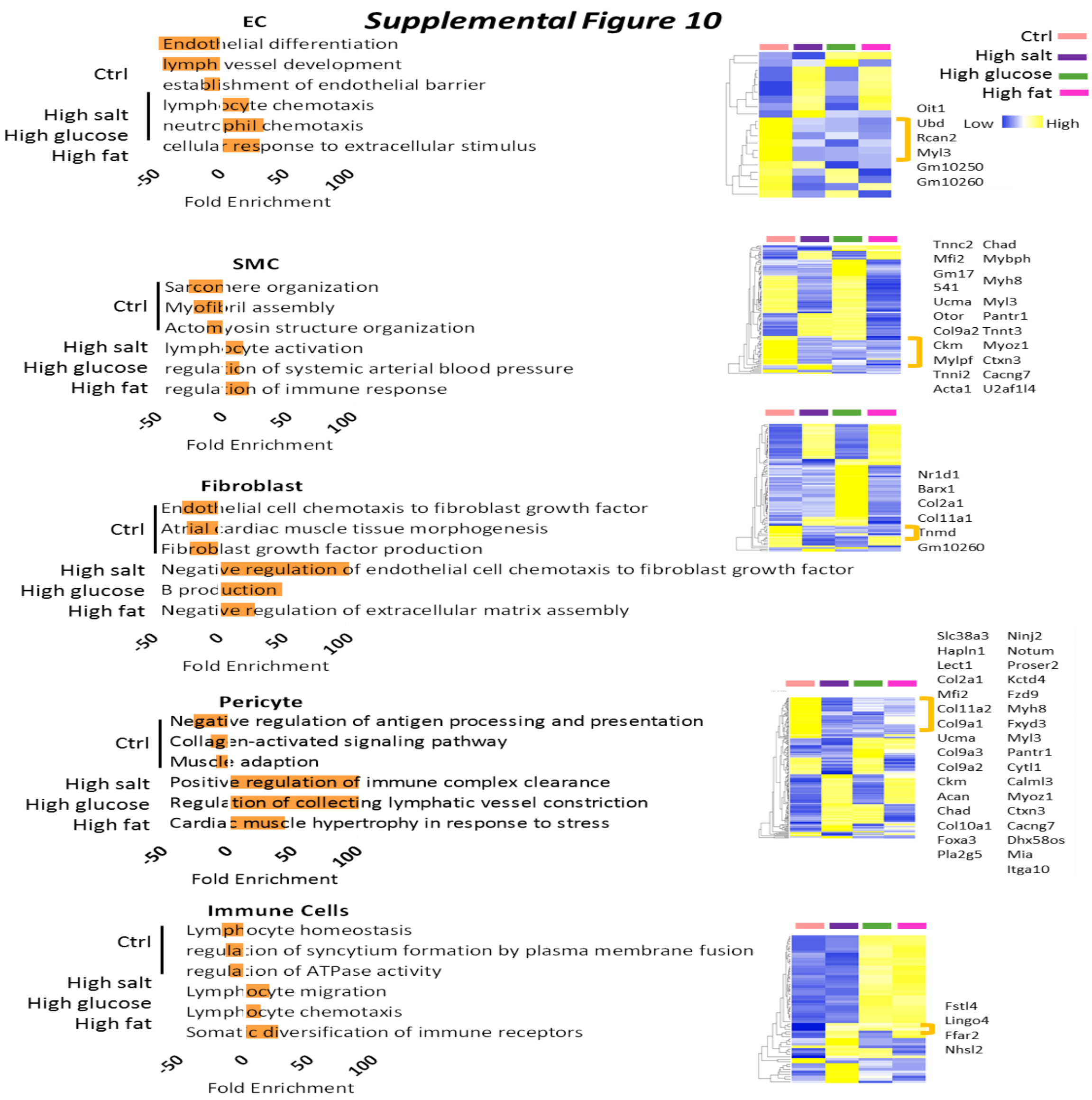

Supplemental Figure 10. Gene expression changes under high-glucose, high-salt, and high-fat conditions.

Left panels: in the indicated cell types, significantly increased or decreased genes after high-glucose, high-salt, and high-fat conditions were enriched for GO biological process. The top 3 increased/decreased biological processes are shown in the bar graphs. Negative fold enrichment values represent decreased GO biological process in control cells. Right panels: commonly changed genes in aortas from mice with a high-salt diet, a high-fat diet, and high plasma glucose. Heatmap showing the gene expression of significantly changed genes (log_2FC >1.5, p <0.01) in the indicated cell types from at-risk aortas. The list of genes on the side are those showing the same tendency of change (down- or up-regulated) in at-risk aortas *vs* healthy controls.

Supplemental Figure 11

a. Subtypes of ECs

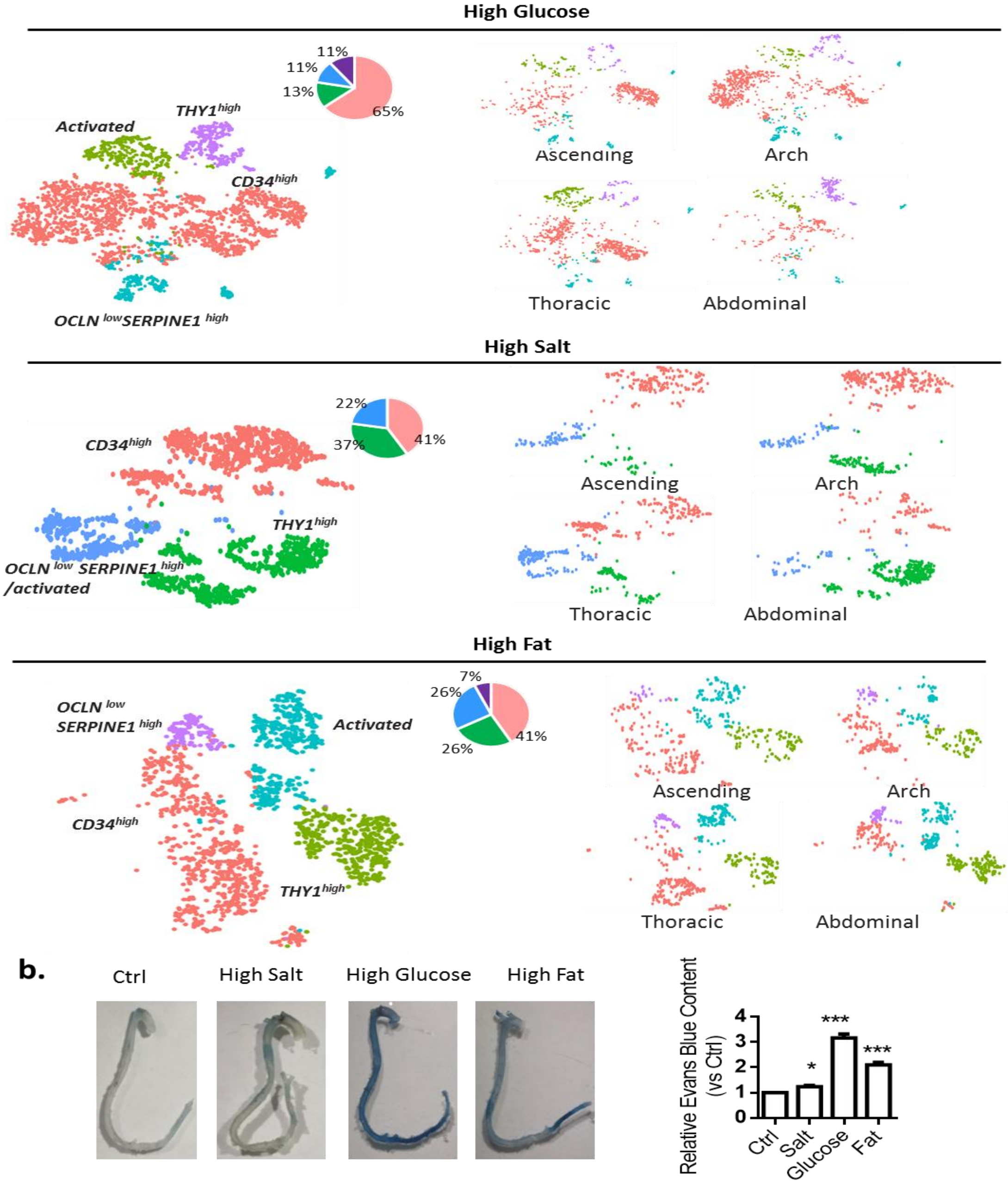

Supplemental Figure 11. Subpopulations of ECs. a, t-SNEs of aortic EC subpopulations, proportions of subpopulations (pie graphs), and regional distribution of subpopulations under high-glucose, high-salt, and high-fat conditions (n = 2 per condition; 3,780, 2,860, and 2,888 cells, respectively). **b,** Aortic permeability under high-glucose, high-salt, and high-fat conditions. Relative content of Evans blue per gram aorta compared to control aorta. The value in control aorta was normalized to 1. *p <0.05, ***p <0.0001 *vs* control.

Supplemental Figure 12

High Glucose

Cell(%)	Ascending	Arch	Thoracic	Abdominal
Activated	13.8	6.2	18.9	16.9
CD34^{high}	65.4	74.9	65.1	38.8
OCLN^{low}SERPINE1^{high}	14.1	10.9	9.1	12.2
THY1^{high}	6.8	7.9	6.9	32.0

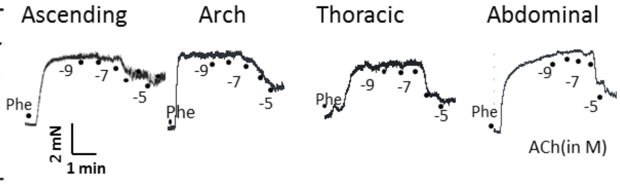

High Salt

Cell(%)	Ascending	Arch	Thoracic	Abdominal
Activated/ OCLN^{low}SERPINE1^{high}	27.3	14.3	19.6	6.9
CD34^{high}	60.6	58.5	64.4	45.0
THY1^{high}	12.1	27.2	16.1	48.1

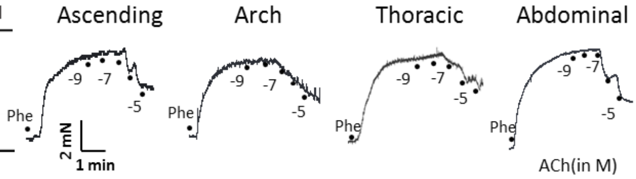

High Fat

Cell(%)	Ascending	Arch	Thoracic	Abdominal
Activated	14.1	10.9	13.1	12.2
CD34^{high}	65.4	74.9	63.1	38.8
THY1^{high}	13.8	6.2	16.9	16.9
OCLN^{low}SERPINE1^{high}	6.8	7.9	6.9	32.0

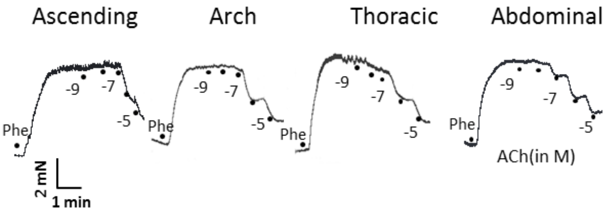

Statistical summary

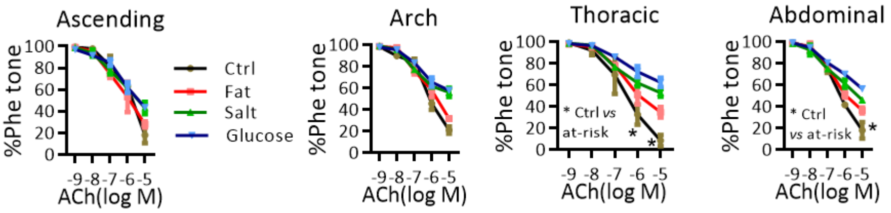

Supplemental Figure 12. **EC composition and vascular dilation in at-risk conditions.** The upper three panels show the percentages of each EC subpopulation and original traces of EC-dependent vascular dilation recorded by wire myography under high-glucose, high-salt and high-fat conditions respectively. The lowest panels are summary of vascular dilation results across aortic segments (compared to Fig. 2f). *p <0.05, control *vs* high fat or high salt or high glucose, two-way ANOVA.

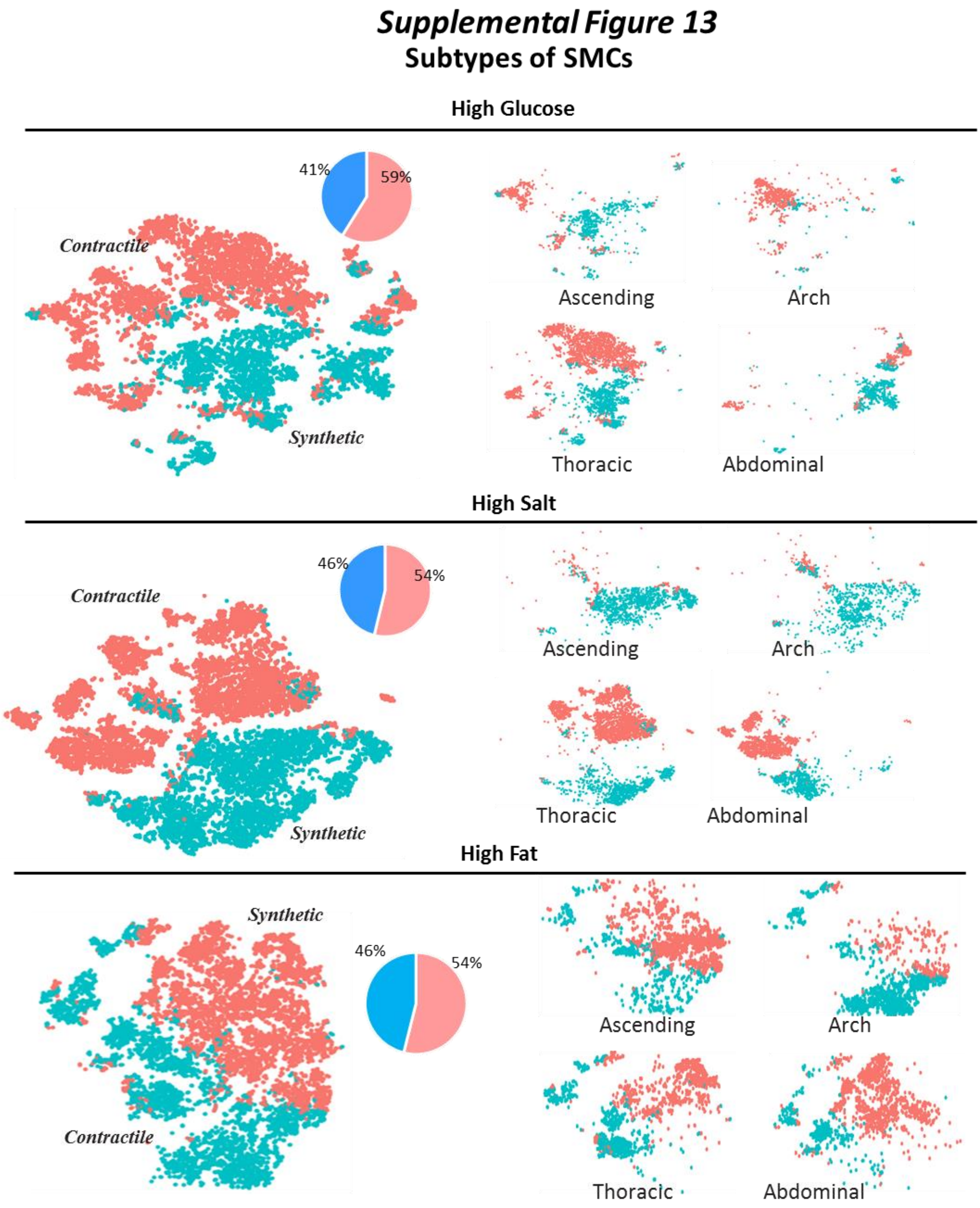

Supplemental Figure 13. Subpopulations of SMCs. t-SNEs of aortic SMC subpopulations, proportions of subpopulations (pie graphs), and regional distribution of subpopulations under high-glucose, high-salt, and high-fat conditions (n = 2 per condition; 8,242, 12,130, and 10,416 cells, respectively).

Supplemental Figure 14

High Glucose

Cell(%)	Ascending	Arch	Thoracic	Abdominal
Contractile	42.4	83.9	65.8	37.4
Synthetic	57.6	16.1	34.2	62.6

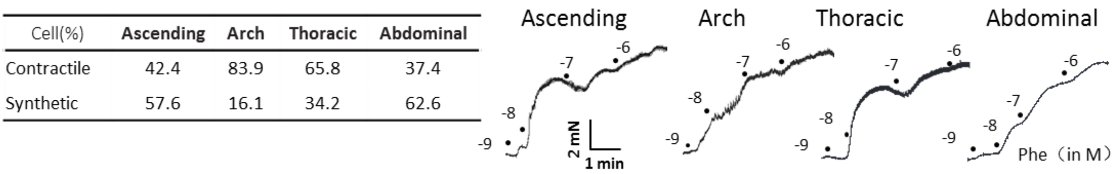

High Salt

Cell(%)	Ascending	Arch	Thoracic	Abdominal
Contractile	14.1	15.1	71.2	65.8
Synthetic	85.9	84.9	28.8	34.2

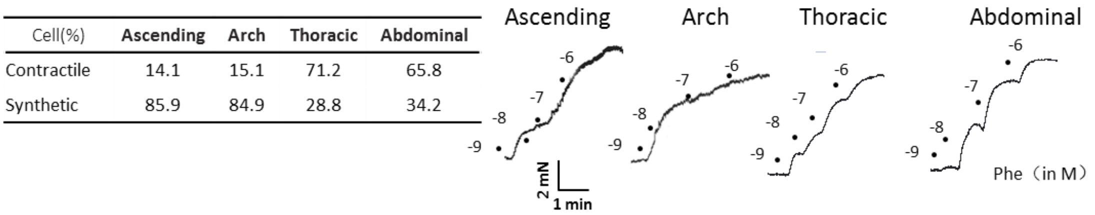

High Fat

Cell(%)	Ascending	Arch	Thoracic	Abdominal
Contractile	73.1	30.6	27.3	48.8
Synthetic	26.9	69.4	72.7	51.2

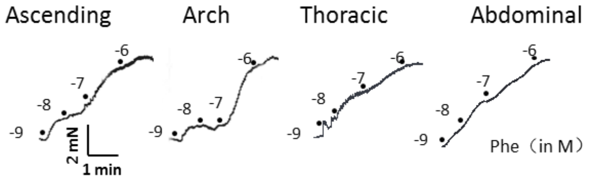

Statistical summary

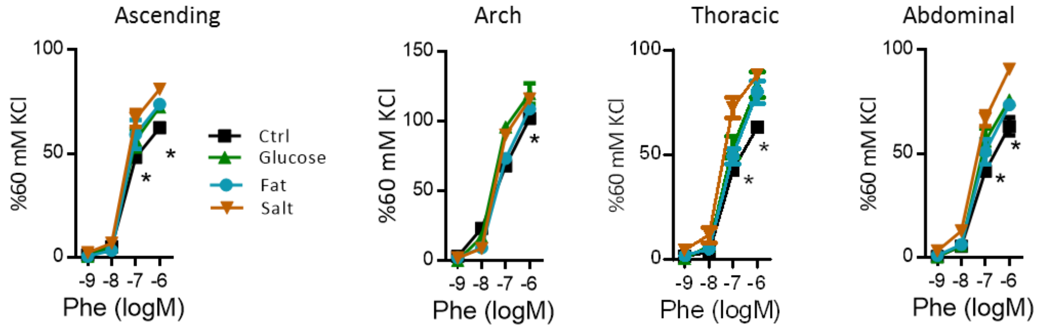

Supplemental Figure 14. SMC composition and vascular contraction in at-risk conditions. The upper three panels show the percentages of each SMC subpopulation and original traces of vascular contraction recorded by wire myography under high-glucose, high-salt and high-fat conditions respectively. The lowest panels are summary of myography results across aortic segments. *p <0.05, control *vs* high fat or high salt or high glucose, two-way ANOVA.

Supplemental Figure 15

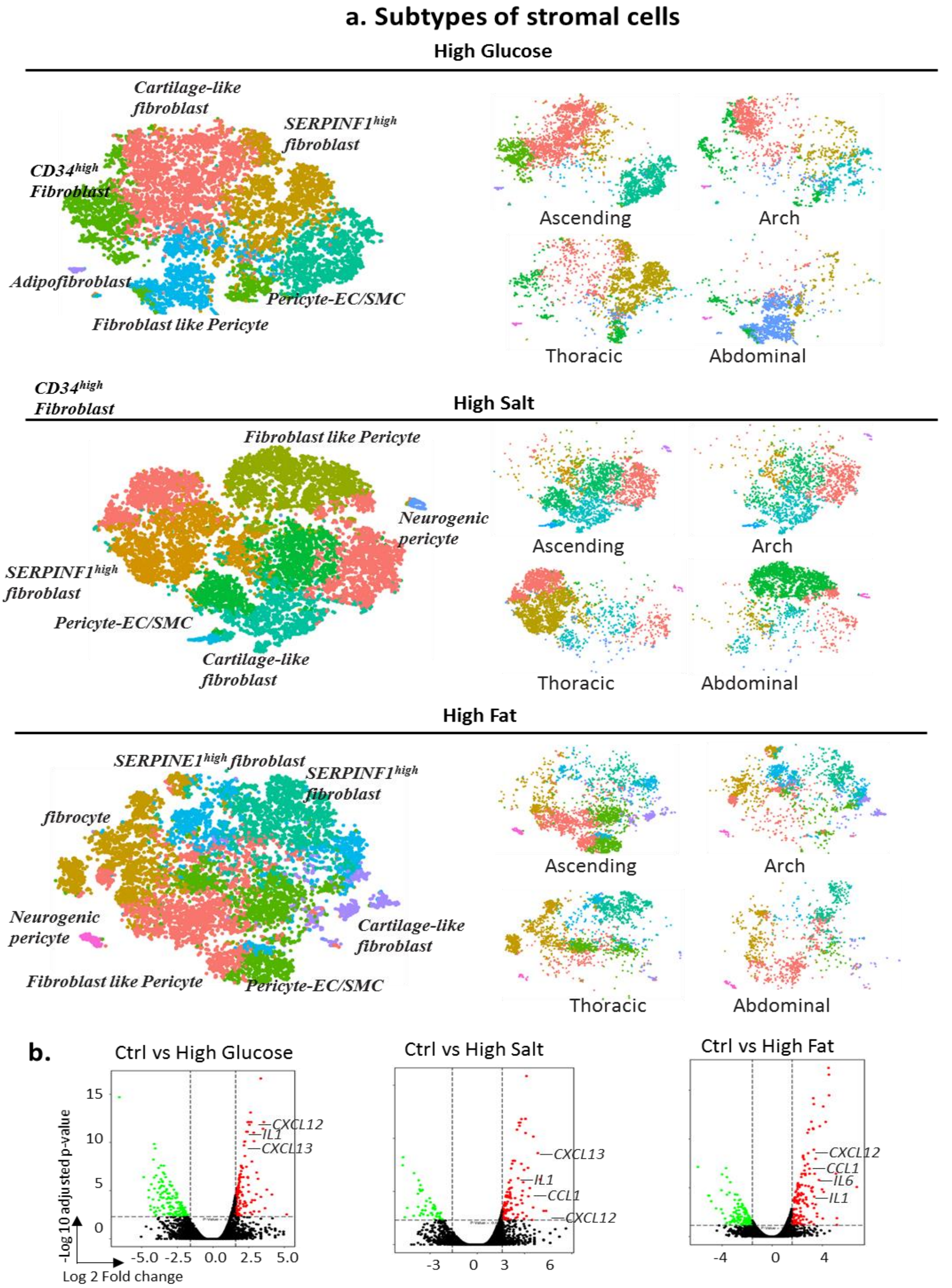

Supplemental Figure 15. Subpopulations of stromal cells. a, t-SNEs of aortic stromal cell subpopulations, and regional distribution of subpopulations under high-glucose, high-salt, and high-fat conditions (n = 2 per condition; 13,130, 15,058, and 12,549 cells, respectively). **b,** Volcano plots showing differentially-expressed genes in fibroblast-like pericytes under high-glucose, high-salt, and high-fat conditions.

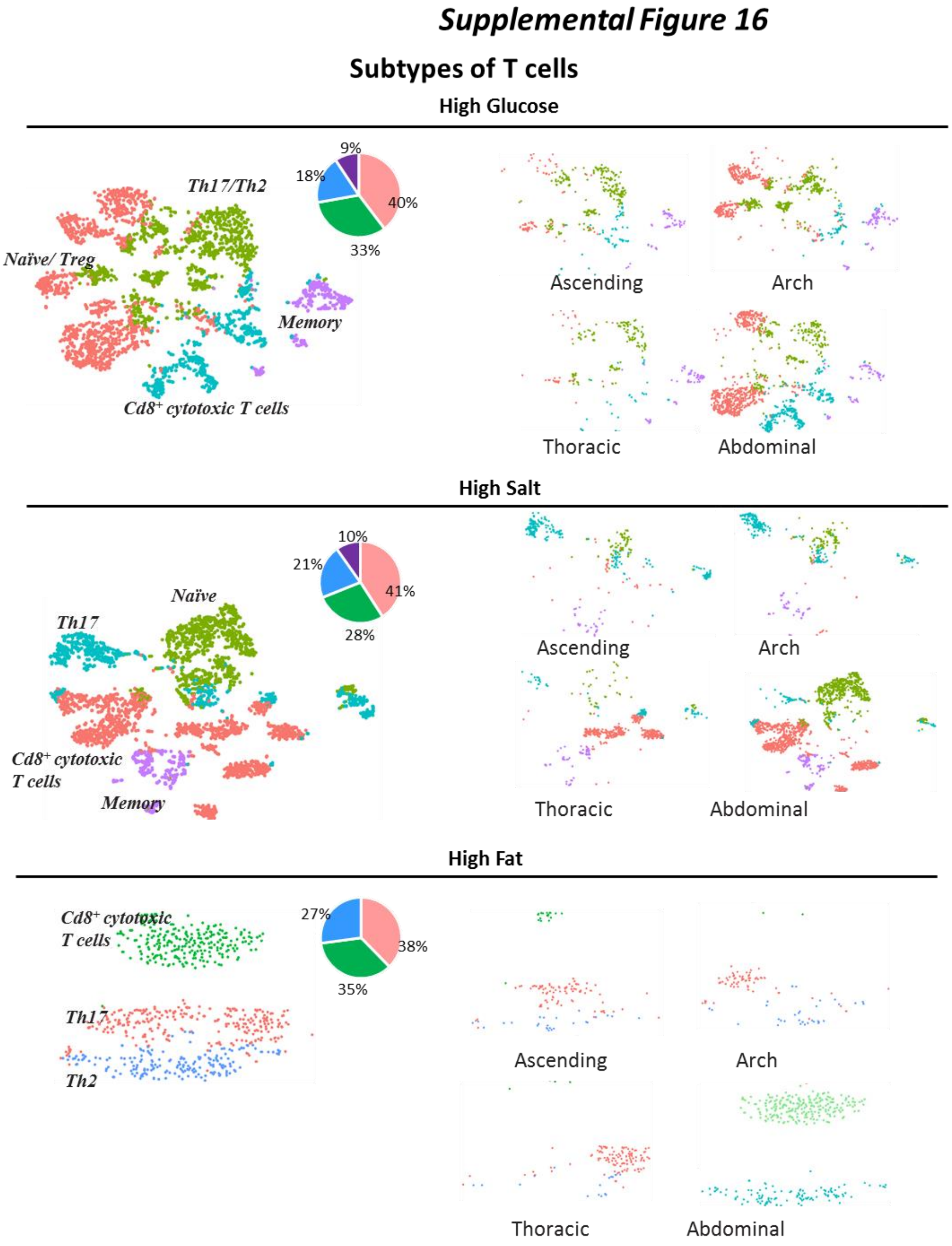

Supplemental Figure 16. Subpopulations of T cells. t-SNEs of aortic T cell subpopulations, proportions of subpopulations (pie graphs), and regional distribution of subpopulations under high-glucose, high-salt, and high-fat conditions (n = 2 per condition; 3,437, 3,305, and 1,444 cells, respectively).

Supplemental Figure 17

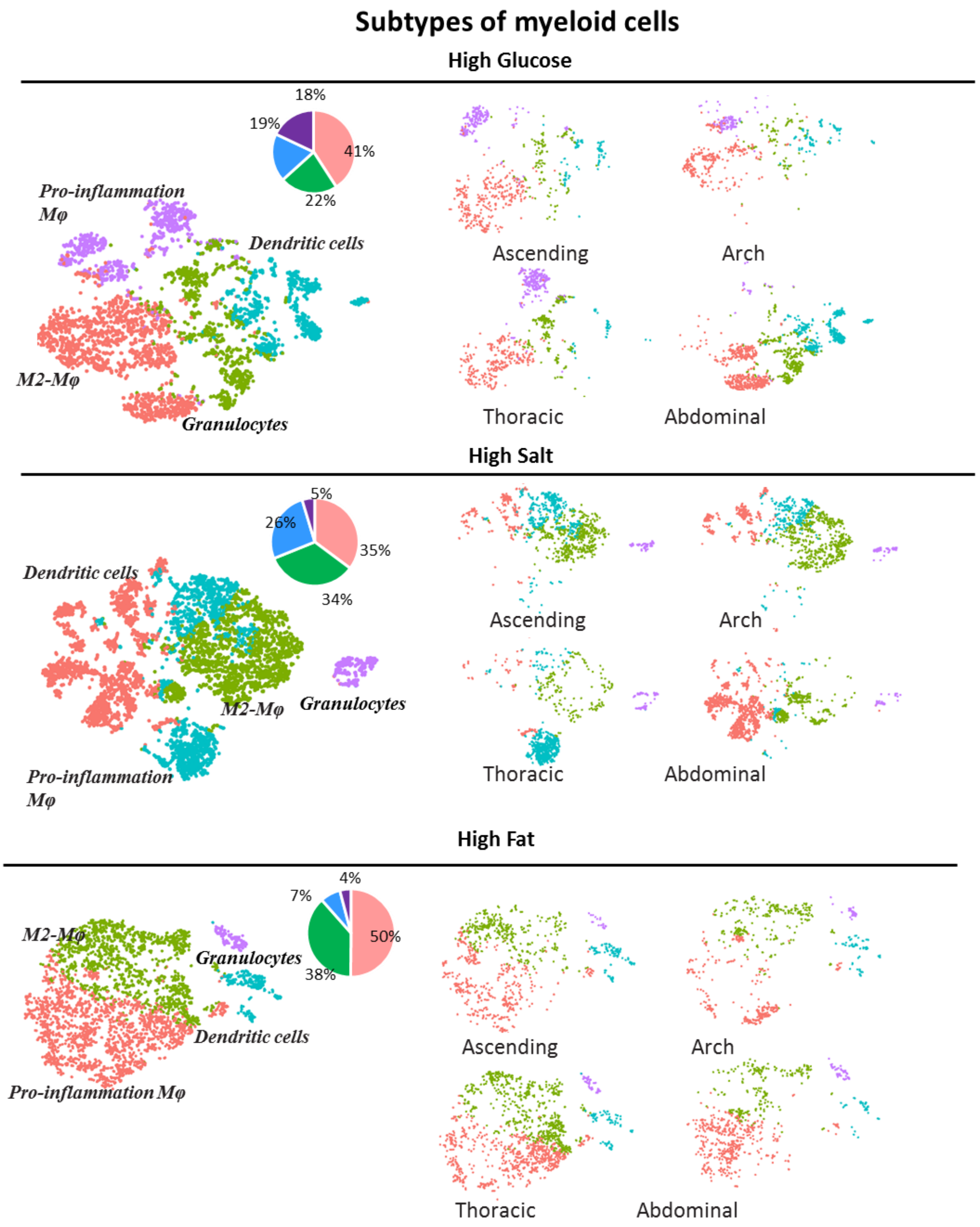

Supplemental Figure 17. Subpopulations of myeloid cells. t-SNEs of aortic myeloid cell subpopulations, proportions of subpopulations (pie graphs), and regional distribution of subpopulations under high-glucose, high-salt, and high-fat conditions (n = 2 per condition; 7,492, 5,669, and 4,267 cells, respectively).

Supplemental Figure 18

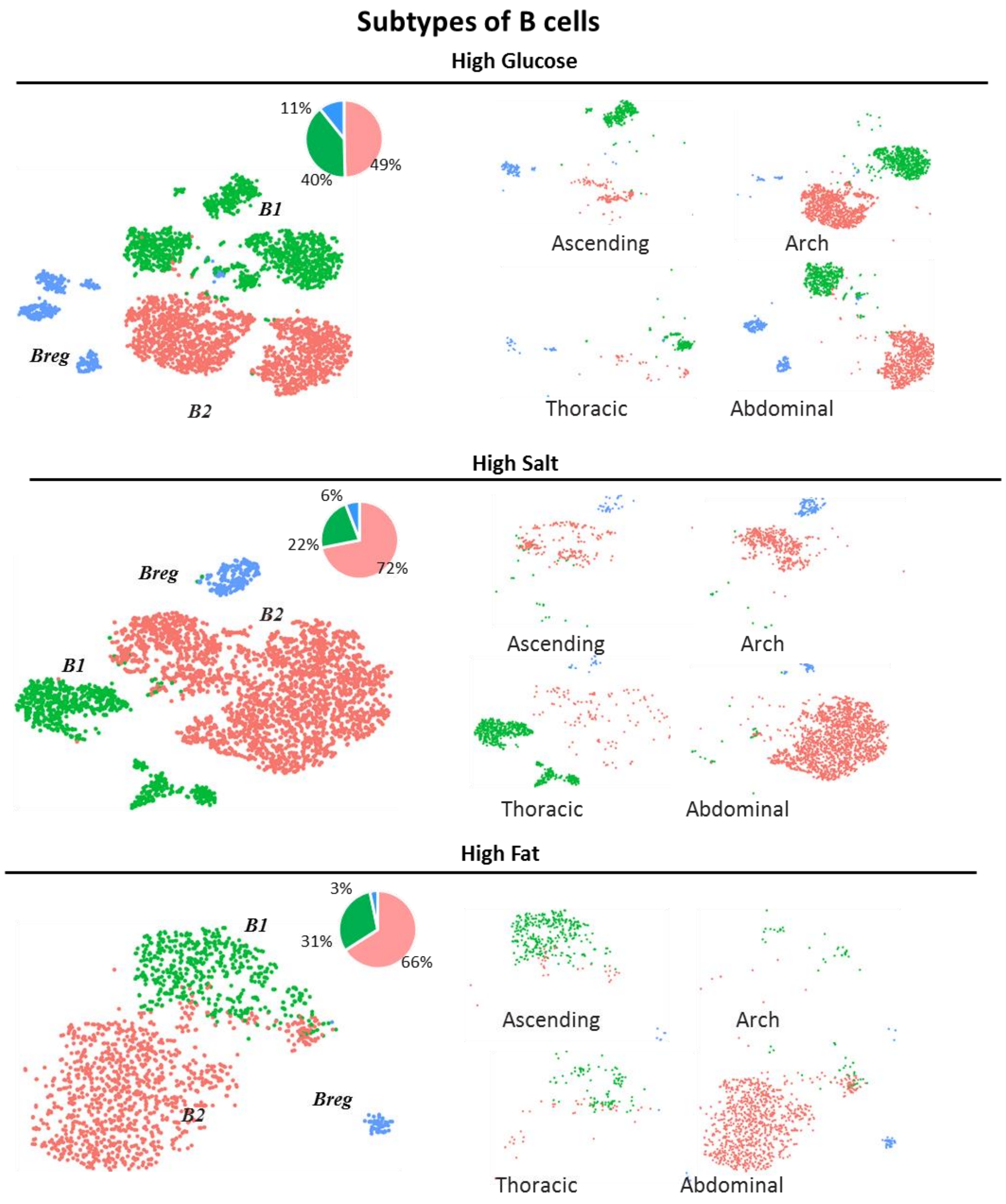

Supplemental Figure 18. Subpopulations of B cells. t-SNEs of aortic B cell subpopulations, proportions of subpopulations (pie graphs), and regional distribution of subpopulations under high-glucose, high-salt, and high-fat conditions (n = 2 per condition; 4,825, 4,034, and 1,678 cells, respectively).

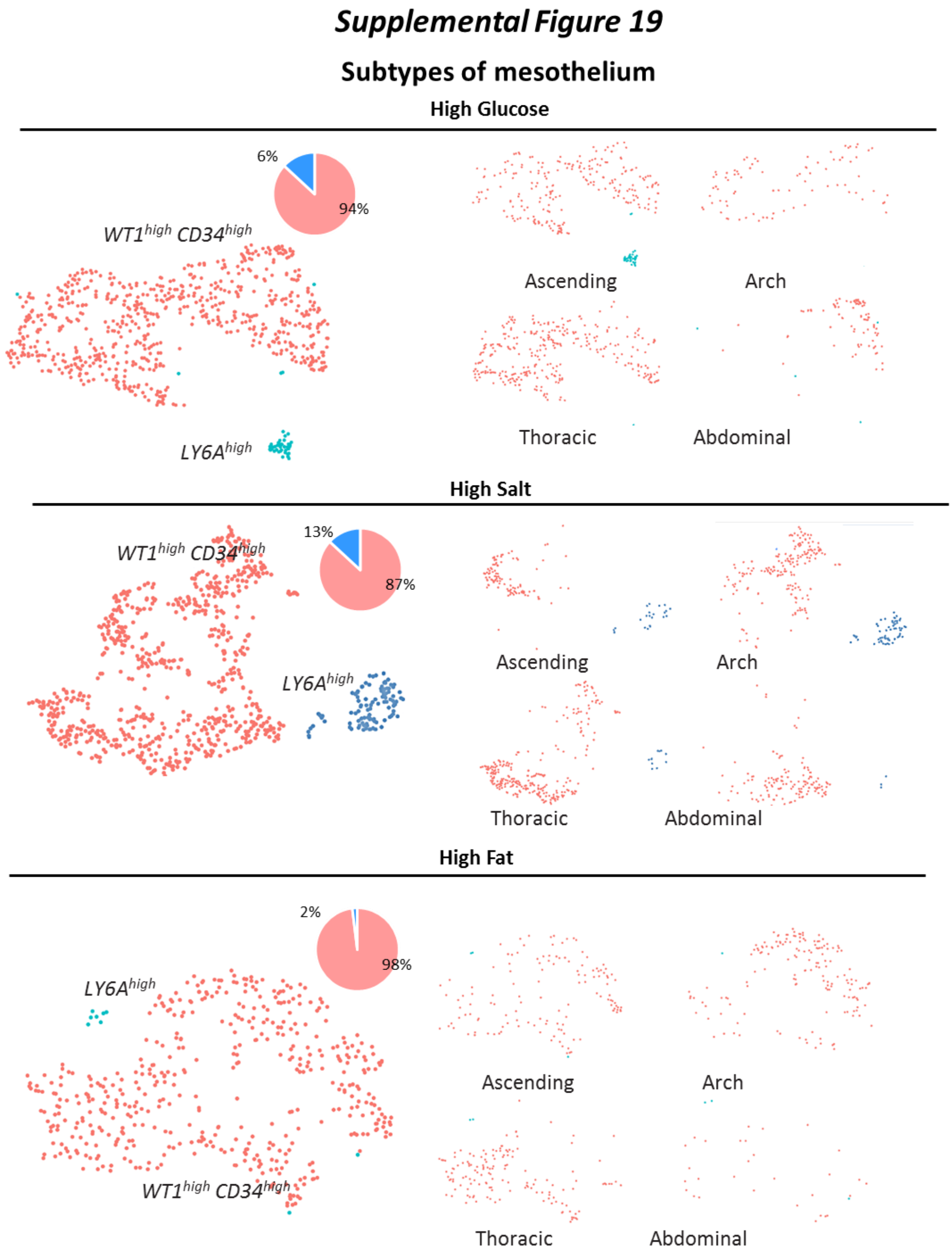

Supplemental Figure 19. Subpopulations of mesothelial cells. t-SNEs of aortic mesothelial cell subpopulations, proportions of subpopulations (pie graphs), and regional distribution of subpopulations under high-glucose, high-salt, and high-fat conditions (n = 2 per condition; 743, 794, and 950 cells, respectively).

Supplemental Figure 20

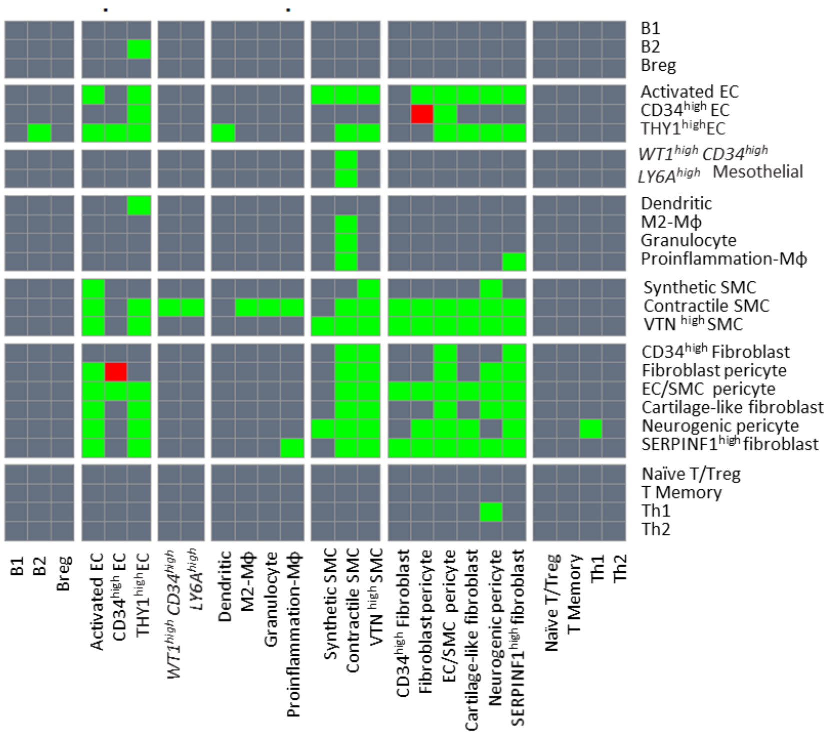

Supplemental Figure 20. Changes in putative ligand and receptor-based cell-cell interaction between subpopulation of aortic cells. scRNA-Seq data of healthy aorta was compared with that of high-salt, high-fat and high-glucose conditions. Red means gain of interaction in all at-risk groups, grey means interaction was not changed in all of the at-risk groups, green means loss of interaction in all at-risk groups,

Supplemental Figure 21

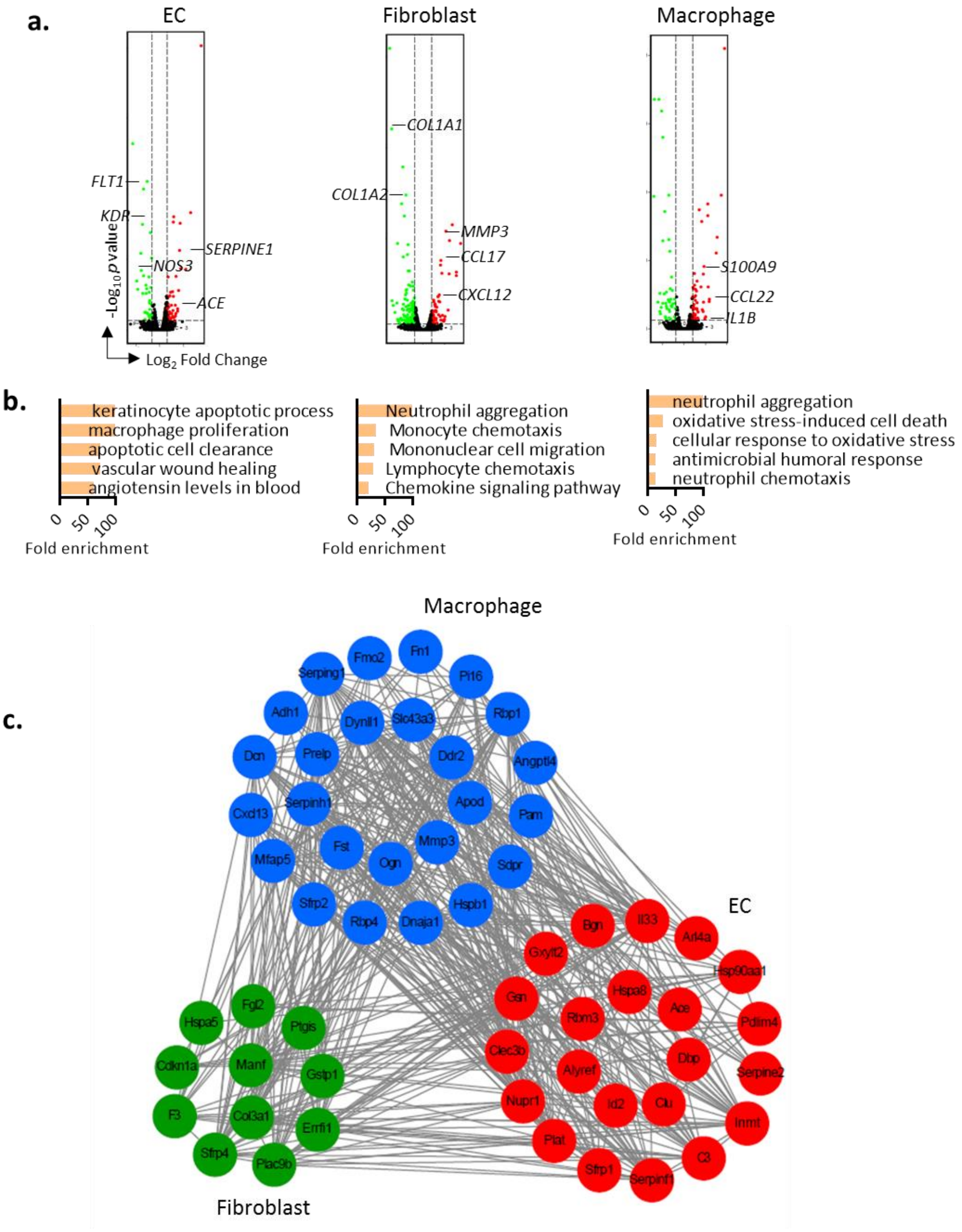

Supplemental Figure 21. a, Volcano plots showing differentially-expressed genes in abdominal ECs, fibroblasts, and macrophages under high-glucose, high-salt, and high-fat conditions *vs* control mice. The genes that commonly changed in high-glucose, high-salt, and high-fat were used for analysis. **b,** GO analysis of differentially-expressed genes. **c,** Gene correlation network among abdominal ECs, fibroblasts, and macrophages during the pathological processes. When the expression of one gene is correlated with that of another (Spearman r >0.75), they are defined as correlated and connected with a black line.
